# Inducible, tightly regulated and growth condition-independent transcription factor in *Saccharomyces cerevisiae*

**DOI:** 10.1093/nar/gku616

**Published:** 2014-07-17

**Authors:** Diana S.M. Ottoz, Fabian Rudolf, Joerg Stelling

**Affiliations:** 1 ETH Zurich and Swiss Institute of Bioinformatics, D-BSSE, Mattenstrasse 26, 4058 Basel, Switzerland; 2 Life Science Zurich PhD Program on Molecular and Translational Biomedicine, Zurich, Switzerland; 3 Competence Center for Systems Physiology and Metabolic Diseases, ETH Zurich, 8093 Zurich, Switzerland

## Abstract

The precise control of gene expression is essential in basic biological research as well as in biotechnological applications. Most regulated systems available in yeast enable only the overexpression of the target gene, excluding the possibility of intermediate or weak expression. Moreover, these systems are frequently toxic or depend on growth conditions. We constructed a heterologous transcription factor that overcomes these limitations. Our system is a fusion of the bacterial LexA DNA-binding protein, the human estrogen receptor (ER) and an activation domain (AD). The activity of this chimera, called LexA-ER-AD, is tightly regulated by the hormone β-estradiol. The selection of the AD proved to be crucial to avoid toxic effects and to define the range of activity that can be precisely tuned with β-estradiol. As our system is based on a heterologous DNA-binding domain, induction in different metabolic contexts is possible. Additionally, by controlling the number of LexA-binding sites in the target promoter, one can scale the expression levels up or down. Overall, our LexA-ER-AD system is a valuable tool to precisely control gene expression in different experimental contexts without toxic side effects.

## INTRODUCTION

Regulated systems allow the adjustment of the expression of target genes by controlling a well-defined input, for example, a chemical. A regulated system should have some important features to make it applicable in multiple experimental setups. First, the system should cover a broad expression range. Second, the system should be tightly regulated to ensure no activity in absence of the input. Third, the activity of the system should not influence metabolism or be influenced by it. Fourth, the input and the activity of the system should not have any toxic effect.

Natural regulated systems (e.g. the galactose- or phosphate-sensitive promoters) have been frequently used to control gene expression in *Saccharomyces cerevisiae*. These promoters are induced by a metabolic signal via a dedicated regulator and the metabolic signal leads to the execution of a specific transcriptional program involving multiple genes (recently reviewed in (1)). By consequence, these systems influence cell growth and their performance strictly depends on the genetic background of the host cell (2). Some natural inducers are toxic (e.g. copper, which regulates the *CUP1* promoter) and they require specific genetic modifications of the host to limit their negative effects (1,3). To overcome the intrinsic limitations of natural regulated systems, heterologous transcription factors have been implemented. A heterologous transcription factor is a fusion of protein domains (DNA-binding domain, activation domain (AD), regulatory domain) isolated from other organisms. Theoretically, such transcription factors do not interfere with the host physiology, as their function is separated from the host metabolism and because they usually depend on inputs that are not metabolized by yeast. In practice, the antibiotic tetracycline is an often used input. It inhibits the popular heterologous transcription factor tTA (2,4), but tTA's mechanism of regulation is not always suitable, as tetracycline needs to be added to the culture medium to keep the system switched off. A reverse tTA mutant that activates transcription upon addition of tetracycline alleviates the problem, yet it has a substantial basal activity (5).

The estrogenic hormone β-estradiol is an interesting input for the regulation of heterologous transcription factors because it ensures tight regulation (6) when binding to the hormone-binding domain of the human estrogen receptor (7). The first β-estradiol-dependent heterologous transcription factor implemented in yeast was a chimera containing the Gal4 DNA-binding domain, the hormone-binding domain of the human estrogen receptor (from now on defined as ER) and the AD VP16 (Gal4-ER-VP16) (8). Contrary to tTA, it is activated upon addition of the input. However, Gal4-ER-VP16 has important limitations. First, the system is toxic when activated with β-estradiol (9). Second, activated Gal4-ER-VP16 also binds to the endogenous *GAL* promoters and thereby influences metabolism. Finally, as the endogenous Gal4 regulator could also bind to the target promoter, this system cannot be used in media containing galactose, unless *GAL4* is knocked-out (9). By substituting the Gal4 DNA-binding domain with artificial zinc finger moieties targeting artificial DNA sequences, a nontoxic, titratable system that does not affect growth rate was obtained (10). However, the system has a low controllability especially in the low-to-intermediate expression range: in this range, the strong AD VP16 needs tight control by β-estradiol, while the use of multiple transcription factor-binding sites to increase expression also increases basal activity with the chosen zinc fingers (11).

We constructed an ER-based heterologous transcription factor that does not show toxic effects, operates predictably in different growth conditions and allows the precise regulation of the expression of the target gene to low, intermediate or high levels. While most engineering efforts for heterologous transcription factors focus on the DNA-binding domain, we emphasize that the selection of the AD is important for system performance by combining experimental analysis and mechanistic mathematical modeling. Specifically, the appropriate choice of the AD is essential to prevent toxic phenotypes and to reach the desired expression levels. By using the bacterial DNA-binding protein LexA, we ensured that our system does not depend on yeast metabolism and therefore we could induce it in different growth conditions. Finally, we could finely modulate the output by multiplying the LexA-binding sites in the synthetic target promoter without changing the basal activity. Overall, our LexA-ER-AD system overcomes the limitations of the previous systems, it displays the essential features needed to precisely control gene expression in multiple experimental setups in yeast, and it illustrates principles of modular construction of heterologous transcription factors.

## MATERIALS AND METHODS

### Plasmid construction

Standard techniques were used for DNA manipulation (12). We used *Escherichia coli* strain DH5α (Life Technologies, Invitrogen, USA) for plasmid preparation. PCR was performed with Phusion High-Fidelity DNA Polymerase (Thermo Scientific, USA), following the manufacturer's instructions. DNA manipulation was performed with restriction enzymes and T4 DNA ligase purchased from New England BioLabs, USA. The reporter and the synthetic transcription factor genes were cloned in integrative vectors derived from the pRS yeast shuttle vector series (13) (Robert Gnügge, unpublished). The *DEG1* termination sequence and the single *lexA* box were cloned by primer annealing and subsequent ligation (14). We multiplied the *lexA* boxes exploiting the flanking compatible restriction sites of AvrII and XbaI. The four transcription factor variants were assembled by isothermal assembly (15). The cassette plasmids for promoter replacement, derived from pBluescript II KS(+), were purchased from Stratagene, USA. The cassette can be amplified with the helper primers forward: 5′-CGAGAGCTTGCCTTGTCCCC-3′; reverse 1 (annealing in the multi-cloning site): 5′-AAGCTTGATATCGAATTCCTG-3′; or reverse 2 (annealing upstream the multi-cloning site): 5′-ATAGAAGTATAGTAATTTATG-3′. All constructs were checked with Sanger sequencing by Microsynth AG, Switzerland. Supplementary Table S1 lists all plasmids used in this work.

### Yeast strain construction

All strains were derived from *Saccharomyces cerevisiae* BY4741 (Euroscarf, Johann Wolfgang Goethe-University Frankfurt, Germany). For integration, plasmids were linearized by restriction enzyme digestion and transformed in yeast using standard methods (16). Transformants were selected with the appropriate selective medium. Integrations were checked by PCR and function. The strains were produced step-wise; we first obtained FRY11 by integrating the constitutively expressed red fluorescence reporter mKate2 in BY4741 using the method described in (17); we derived all the other strains of this work from FRY11; we integrated the transcription factor variants in the *his3Δ1* locus, and the reporter genes into the *ura3Δ0* locus. For the *URA3* promoter replacement experiment, we first reconstituted the wild-type *URA3 locus* by transformation with a PCR product containing the gene. We used the primers 5′-actgcacagaacaaaaacctgcaggaaacgaagataaatcCGAGAGCTTGCCTTGTCCCC-3′ and 5′- gatgagtagcagcacgttccttatatgtagctttcgacatAAGCTTGATATCGAATTCCTG-3′ to amplify the cassette and transformed. After transformation, we checked the *lexA* boxes copy number with 5′-GTCGATTCGATACTAACGCC-3′ and 5′-ATAGAAGTATAGTAATTTATGCTGCAAA-3′. Supplementary Table S2 lists all yeast strains used in this work.

### Media

All chemicals, unless stated, were purchased from Sigma-Aldrich Co., Germany. We prepared yeast media as described in (18). For all experiments, we grew the yeast strains in synthetic media. Synthetic media (S) contained 0.17% yeast nitrogen base without amino acids or ammonium sulphate (BD Biosciences, Germany), 0.5% ammonium sulphate as nitrogen source and 2% glucose (D) or glycerol (Gly) as carbon source. SDC and SGlyC media contained a complete complement of amino acids and nucleotides (C). SDP medium contained 1 mg/ml proline (P) as unique nitrogen and amino acid source. For yeast strain transformation, we grew cells at 30°C in YPD, containing 1% yeast extract (BD Biosciences, Germany), 2% peptone (BD Biosciences, Germany) and 2% glucose. Transformants were isolated in appropriate selective SD medium by auxotrophy complementation. For induction experiments, we diluted β-estradiol from a 10 mM stock in 100% ethanol.

### Flow cytometry

We precultured the strains overnight at 25°C. We diluted the overnight cultures in fresh synthetic medium and grew them to a density of 4.0 × 10^6^ cells/ml. At time 0, we aliquoted 500 μL of each culture in 2.5 ml 96-squared-well plates (HJ-Bioanalytik GmbH, Germany) and induced with a concentration series of β-estradiol. During the induction, cells were cultured at 25°C and vigorously shaken (19,20). We kept the cultures in exponential phase by diluting them regularly in fresh medium.

At each time point, we took 100 μL of culture from each well and treated it with 1:1000 cycloheximide, from a stock of 70 mg/ml in 100% dimethyl sulfoxide (DMSO, Sigma-Aldrich Co., Germany) to stop translation. We incubated the samples at room temperature for 30 min in order to allow fluorescence protein maturation.

We subsequently analysed the samples with an LSRFortessa^TM^ LSRII cell analyser coupled with a high-throughput sampler (HTS) from BD Biosciences, Germany. To measure Citrine yellow fluorescence, we used a 488 nm excitation laser (100 mW) and a 530/30 nm emission filter. To measure mKate2 red fluorescence, we used a 561 nm excitation laser (100 mW) and a 610/20 nm emission filter. We used the FACSDiva software (BD Biosciences, Germany) for data recording. For each sample, we recorded 10 000 events.

We used the software R (R Core Team 2013, Vienna, Austria) with the Bioconductor package (http://www.bioconductor.org) to analyse the flow cytometry data. We gated for un-budded cells, which were identified by analysing the signal width of the forward scatter and the side scatter (FSC-W/SSC-W) plots as shown in Supplementary Figure S1. After gating, we obtained approximately 2000–4000 events for each sample analysed.

### Western blots

We precultured the strains overnight at 25°C in SDC. We diluted the overnight cultures in fresh medium and grew them to a density of 2.2 × 10^7^ cells/ml. At time 0, we induced the cultures with β-estradiol.

We aliquoted the cultures in 5 ml volumes and added tricholoroacetic acid (Sigma-Aldrich Co., Germany) to a final concentration of 5%.

We extracted total protein, separated it by SDS-PAGE and transferred it onto a membrane as described in (12). When detecting Citrine and β-actin, we used a nitrocellulose membrane (GE Healthcare Europe, Switzerland). When detecting LexA, we used a polyvinylidene fluoride membrane (Merck Millipore KGaA, Germany).

We used a mouse primary antibody for LexA from Dualsystems Biotech AG, Switzerland (catalogue number P06004). We diluted anti-LexA 1:5000 in phosphate buffered saline + 0.1% Tween 20 (PBS-T). To detect Citrine, we used a mouse primary antibody for GFP from F. Hoffmann- La Roche Ltd, Switzerland (cat. 11814460001). We diluted anti-GFP 1:1000 in PBS-T + 0.5% bovine serum albumin (BSA, from Serva Electrophoresis GmbH, Germany). As a loading control, we detected β-actin with anti-β-actin mouse antibody from Abcam, UK (catalogue number mAbcam 8224) diluted 1:2000 in PBS-T. The secondary antibody was an anti-mouse from sheep linked to the horseradish peroxidase from GE Healthcare Europe GmbH, Switzerland (catalogue number na931v) diluted 1:10000 in PBS-T + 1% milk (AppliChem GmbH, Germany).

Detection was performed with ECL Detection Reagents from GE Healthcare Europe GmbH, Switzerland. We used Super RX-Films from Fujifilm AG, Switzerland, for imaging.

### Growth curves

We precultured the strains overnight at 30°C in SDC. We then diluted the cultures and grew them to a density of 1.5 × 10^6^ cells/ml.

In each well of a 96-well plate (BD Labware, USA), we inoculated 10 000 cells in 200 μL of fresh medium with a defined β-estradiol concentration. Per each strain of β-estradiol concentration combination, we prepared triplicates.

We incubated the plate in the TECAN M200 reader (Tecan group Ltd., Switzerland) at 30°C for 55 h. The plate was shaken all the time. Absorbance at 600 nm was measured every 6.5 min.

We plotted the growth curves using the software R. For each time point, we calculated the mean of the absorbance at 600 nm and the standard deviation of each triplicate.

### Quantitative real-time PCR

We precultured the strain overnight at 25°C in SDC. We diluted the overnight cultures in fresh medium and grew them to a density of 1.0 × 10^7^ cells/ml. At time 0, we induced the culture with 2000 nM β-estradiol. At each time point, we took 2 ml from the culture, pelleted the cells by centrifugation and snap-froze the pellets in liquid nitrogen. We performed three independent biological replicates.

We used the MasterPure^TM^ Yeast RNA Purification kit from Epicentre Biotechnologies, USA, to extract total RNA. We checked the RNA quality by agarose gel electrophoresis, as described in (12). We measured the concentration of our RNA samples with the NanoDrop 2000c (Thermo scientific, USA).

We converted RNA into cDNA with the First Strand cDNA Synthesis kit from Fermentas, Lithuania, using the oligo(dT)_18_ primers provided by the kit.

We performed quantitative real-time PCR (qRT-PCR) on a LightCycler^®^ 480 Instrument using the LightCycler^®^ 480 DNA SYBR Green I Master kit (F. Hoffmann- La Roche Ltd, Switzerland). The PCR protocol had a first denaturation step (95°C for 10 min), followed by 45 cycles of 10 s at 95°C, 10 s at 58°C and 10 s at 72°C. We measured Citrine and *ACT1* cDNAs. We performed three technical replicates for each biological sample.

We used the second derivative maximum method of the LightCycler^®^ 480 Software (F. Hoffmann- La Roche Ltd, Switzerland) to calculate the C_P_ values. Relative quantification of Citrine expression in comparison to *ACT1* was performed as described in (21). For each time point per each biological replicate, we calculated the mean and the standard deviation of the technical triplicates.

For Citrine we used the primers 5′-GGTTGAATTAGATGGTGATGTTA-3′ and 5′-GGCAATTTACCAGTAGTACAAA-3′ that amplified a DNA stretch of 117 nucleotides starting from nucleotide number 69 of the Citrine ORF. For *ACT1* we used primers 5′-CAGGTATTGCCGAAAGAA-3′ and 5′-CCACATTTGTTGGAAGGTA-3′ that amplified a DNA stretch of 130 nucleotides starting from nucleotide number 1229 of the *ACT1* ORF.

### Spot assay

We precultured the strains overnight at 30°C in SDC. We then diluted the cultures and grew them to a density of 8.0 × 10^6^ cells/ml.

We spotted 10 000 cells and two 1:10 dilutions on SDC, SD medium lacking uracil (SD-URA) and SD-URA + 2000 nM β-estradiol plates. We incubated the plates at 30°C overnight.

### Mathematical modeling

We developed a mechanistic dynamic model in the form of 29 ordinary differential equations (ODEs) based on the network of elementary reactions compiled in Supplementary Table S3 and on mass-action kinetics. All model simulations and analysis were performed in Matlab R2013b (Mathworks, Natick, MA, USA); Supplementary Table S4 provides the corresponding initial conditions. Key model components are transcription factor (TF), hormone, inhibitory protein (which retains the TF in the cytoplasm when unbound to hormone), RNA polymerase II (which has a dual role in the model, namely to effect gene expression and to serve as a cellular resource on which growth depends to capture toxicity phenomenologically) and the corresponding mRNAs and genetic elements (operators and coding regions). Beyond binding/unbinding events and diffusive transport between nucleus and cytoplasm, gene expression and its (potentially autocatalytic, in the case of RNA polymerase II) control are included for all components where relevant.

To generate the system of ODEs from the biochemical reaction set as given in Supplementary Table S3, we consider the mass balances of *n* individual components in a biochemical network with *r* reactions. The general form of such an ODE system is:

\begin{equation*} \frac{{d{\bf c}(t)}}{{dt}} = {\bf N} \cdot {\bf v}\left( {{\bf c}(t),{\bf u}(t),{\bf k}} \right),\quad {\bf c}(t_0 ) = {\bf c}_0 \end{equation*}

with the *n x 1* vector of time-dependent concentrations **c**(t), the *n x r* stoichiometric matrix **N** whose entries are given by the molecularities of reaction educts and products, and the *r x 1* vector function of reaction rates - or fluxes- **v**(?) that are calculated assuming mass-action kinetics. The fluxes depend on the system state **c**(t), on potentially time-varying inputs **u**(t) such as the β-estradiol concentration in the medium and on kinetic parameters **k** such as affinity constants. Finally, **c**_0_ denotes the initial state of the system, for instance, absolute protein concentrations.

In addition to mass-action kinetics, the model contains three auxiliary functions (see Supplementary Table S5 for parameter definitions): (i) a growth function $\mu = \mu _{\max } \cdot \frac{{Pol_{total}^{N_{HMU} } }}{{K_{MMU}^{N_{HMU} } + Pol_{total}^{N_{HMU} } }}$ that models the specific growth rate as a Hill-type function of the (experimentally determined, condition-dependent) maximal specific growth rate $\mu _{\max }$, the total polymerase concentration $Pol_{total}^{}$ and the corresponding Michaelis–Menten and Hill coefficients; (ii) a function $N_{OP} (p_{TF} )$ for determining the probability of polymerase binding to transcription factors bound to *lexA* boxes, where the probability of occupancy of *lexA* boxes, $p_{TF}$, is calculated from the corresponding system states; assuming that steric limitations allow only a single polymerase molecule to bind simultaneously, the effect of steric limitations is captured by a binomial-like distribution $N_{OP} (p_{TF} ) = p_{TF} \cdot (1 - p_{TF} )^{n - 1}$ where $n$ is the number of *lexA* boxes and (iii) a general translation rate constant $k_{TL}$ that incorporates the correlation between specific growth rate and ribosome abundance by quadratic interpolation of the corresponding data in (22) and normalizing to growth on glucose.

To map the model quantities to experimental data, we included two scaling factors (for fluorescence and mRNA abundance) as well as the following measurement models. Linear regression on variance (s.d.) as a function of average fluorescence signal from flow cytometry yielded an estimated lower detection limit of 2037 A.U.; with the corresponding slope, we assigned a variance of 2344 A.U. to all data points below this limit (Supplementary Figure S2). To estimate variances for PCR data, we employed error propagation on the raw data. We assumed 10% measurement error for specific growth rates.

The experimental data were partitioned into a training set that included only constructs with four *lexA* boxes grown in SDC (fluorescence dynamics, Figure 2; mRNA dynamics, Supplementary Figure S3; specific growth rates, Figure 4B) and a validation set with all other data (growth conditions, Supplementary Figure S4; variable number of *lexA* boxes, Supplementary Figures S5 and S6). We used the training data to estimate 45 model parameters that could not be determined *a priori* (see Supplementary Table S4 for details). For global optimization, we employed an evolutionary strategy (23) to minimize the weighted least-squares objective function

\begin{equation*} \chi ^2 ({\bf k }) = \sum\limits_{i = 1}^{n_e } {\frac{1}{{n_i }}\sum\limits_{j = 1}^{n_i } {\left( {\frac{{c_{ij}^m - c_{ij}^c ({\bf k })}}{{\sigma _{ij} }}} \right)^2 } } \end{equation*}

where **θ** is the vector of model parameters, *j* is the index for the data points in experiment *i* (*n_e_* experiments with *n_i_* data points in experiment *i*), **c^c^** are the calculated state variables (concentrations) of the ODE model, **c^m^** is the vector of experimental data, and σ*_ij_* is the corresponding measurement variance (s.d. according to the measurement models above). Simulations for the validation data used the estimated parameter values, except for condition-specific parameters. We used a χ^2^-test to evaluate model fit, with χ^2^ = 101.2, 45 estimated parameters and 401 degrees of freedom for the training data and χ^2^ = 860.5, 1014 d.o.f. for the validation data.

**Figure 1.**
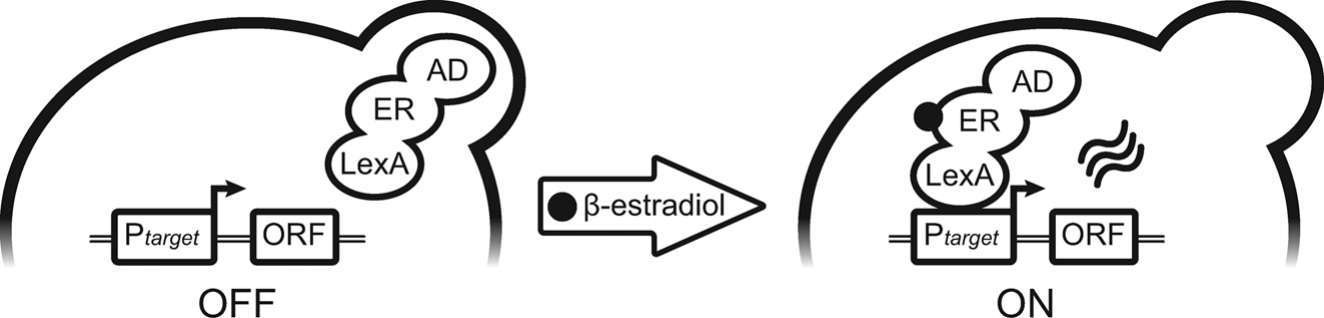
The mechanism of action of the LexA-ER-AD system. ER: hormone-binding domain of the human estrogen receptor; AD: activation domain; P*target*: target promoter; ORF: open reading frame.

**Figure 2.**
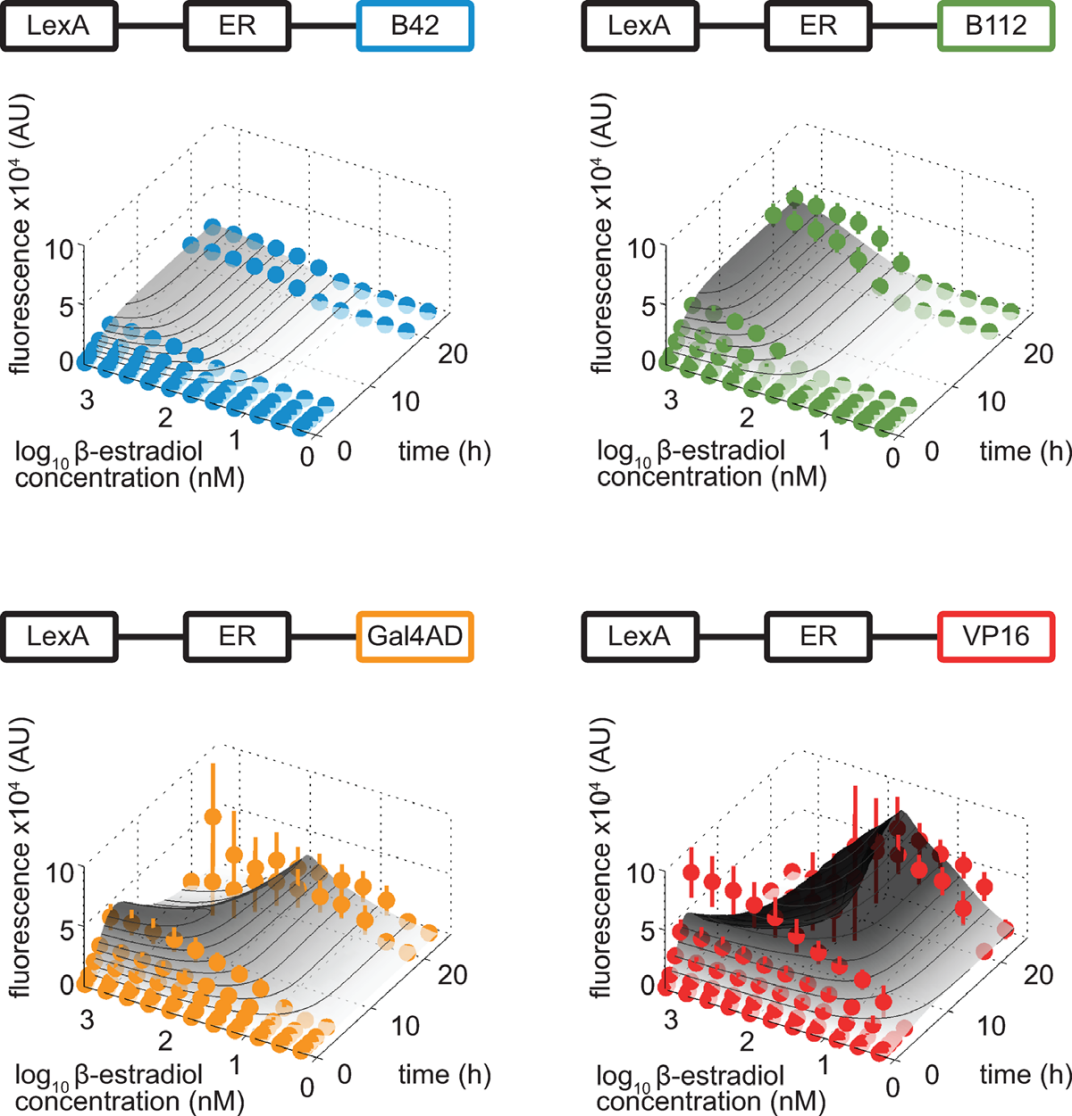
Time course of the titration of LexA-ER-AD activity. Strains containing LexA-ER-B42, LexA-ER-B112, LexA-ER-Gal4AD or LexA-ER-VP16 and a target promoter with four *lexA* boxes driving Citrine expression (FRY418, FRY666, FRY667, FRY743) were incubated in a concentration series of β-estradiol in SDC. At each time point, the induction levels were measured by flow cytometry. Experimental (symbols, mean ± standard deviation) and simulated (gray surfaces and contour lines) time-dependent dose-response are plotted against the logarithmic concentration of inducer.

**Figure 3.**
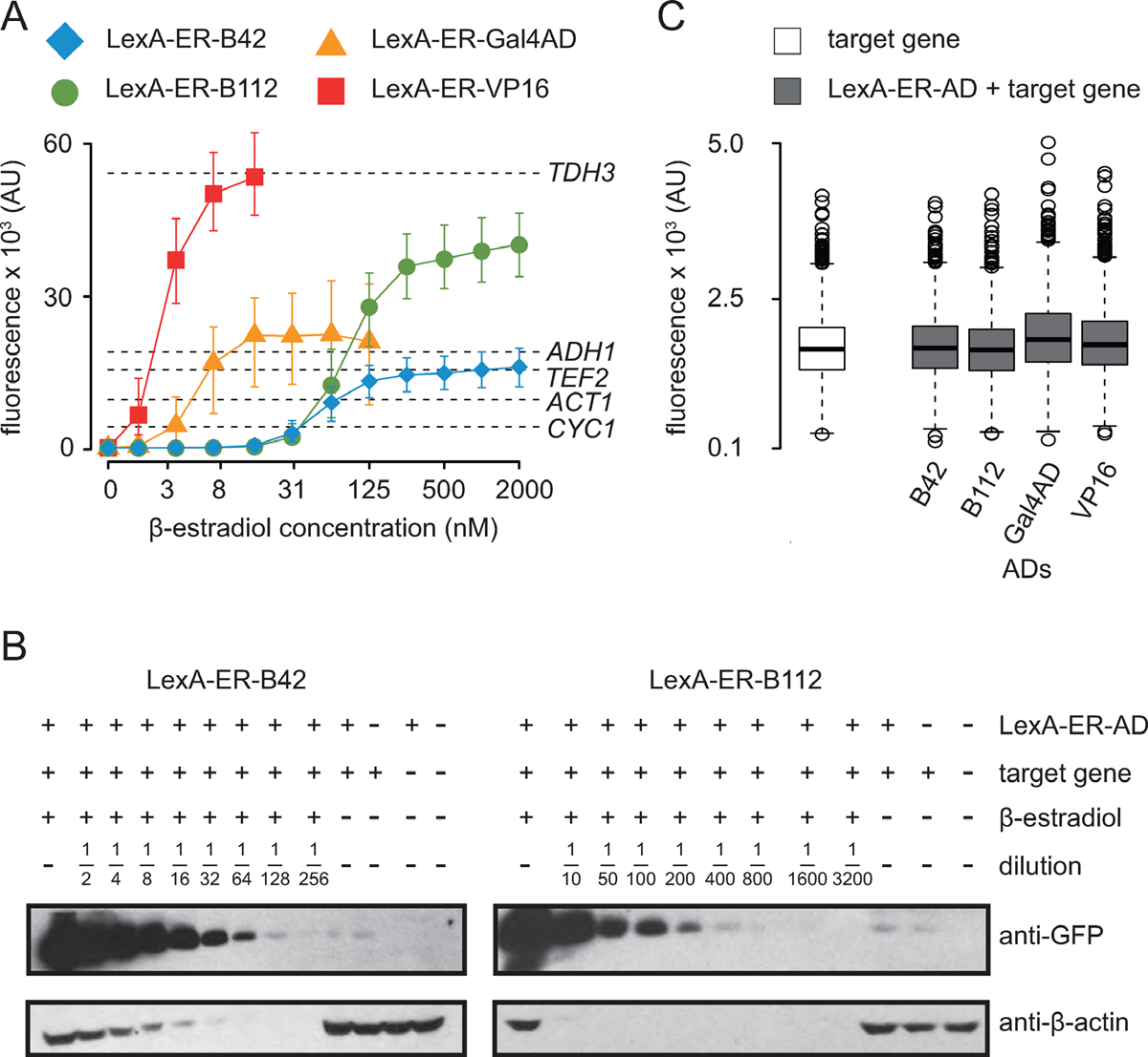
Induction levels and tight regulation of LexA-ER-AD. (**A**) The expression levels of constitutive promoters were plotted on top of the LexA-ER-AD 24-h titration curves obtained by flow cytometry shown in Figure 2. We only considered the concentration ranges of β-estradiol in which our expression system reached a steady state. The x-axis is logarithmic. Symbols represent the median and error bars the 25th and the 75th percentiles of the fluorescence signal (area) distribution measured by the cytometer. Each dashed horizontal line represents the median of the yellow fluorescence signal distributions obtained by expressing Citrine from the constitutive promoters indicated on the right side of the graph (FRY744, FRY745, FRY746, FRY748 and FRY757). (**B**) Western blots to determine protein induction fold by LexA-ER-B42 (left) and LexA-ER-B112 (right) upon incubation with 2000 nM β-estradiol in SDC for 24 h (strains: FRY418 and FRY667). Citrine levels were assayed using an anti-GFP antibody. As loading control, we detected the β-actin with an anti-β-actin antibody. The induced samples were diluted as indicated. As controls, we loaded the un-induced strains (FRY418 and FRY667), a strain bearing only the target gene (Citrine under the control of four *lexA* boxes, FRY484), a strain bearing only the transcription factor (FRY312) and an ‘empty’ strain (FRY11). (**C**) Flow cytometry of the basal activity of LexA-ER-AD. Cells were cultivated in SDC lacking β-estradiol. The target gene strain contained only the target gene with four *lexA* boxes in its promoter (FRY484); the LexA-ER-AD + target gene strains contained both transcription factor and target gene (FRY418, FRY666, FRY667 and FRY743). The LexA-ER-AD variant is indicated under each boxplot, which summarizes the distribution of the fluorescence signal (height) measured.

**Figure 4.**
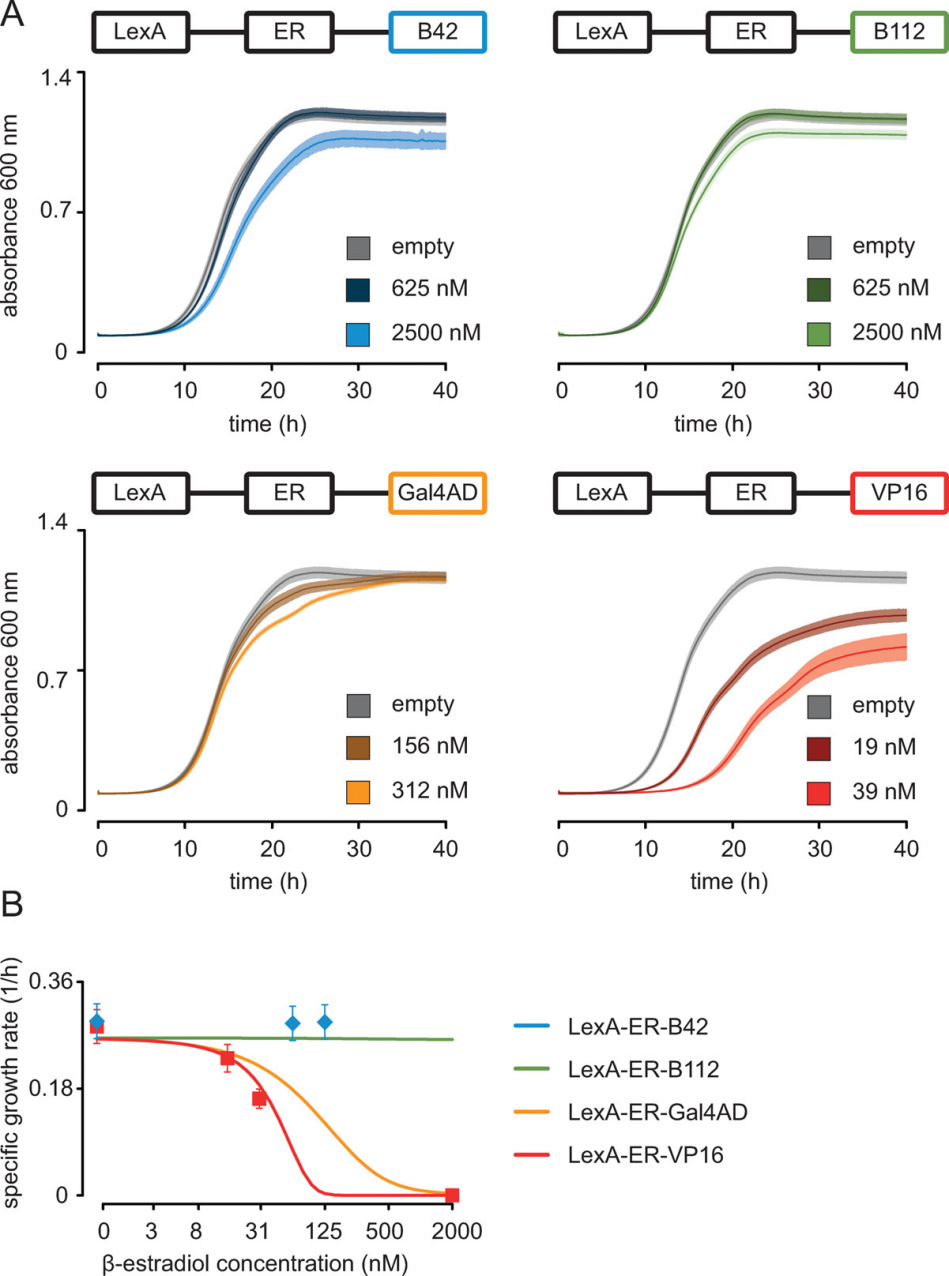
Effects of LexA-ER-AD activation on cell growth. (**A**) Strains containing a LexA-ER-AD variant and no target gene (FRY312, FRY460, FRY544 and FRY758) were induced with variable amounts of β-estradiol in SDC. In each panel, we plotted two growth curves of a LexA-ER-AD variant incubated with two different β-estradiol concentrations. As control, we plotted an ‘empty’ strain (FRY11) grown in 2500 nM β-estradiol. For each curve, we plotted the mean of triplicates (in full color), and ± standard deviation (in semi-transparent color). (**B**) Experimental (symbols; exponential growth rate with mean and ± standard deviation) and predicted (lines; 5 h after induction by β-estradiol) dose-response curves for the specific cellular growth rate, representing dose-dependent toxicity of the individual constructs.

## RESULTS

### The LexA-ER-AD system

Our synthetic transcription factor LexA-ER-AD is a fusion of three domains: the bacterial DNA-binding protein LexA (1–202 aa) (24), the hormone-binding domain of the human estrogen receptor (ER) (282–595 aa) (7,8) and an AD (Figure 1). The ER confers regulation to our system by binding the estrogenic hormone β-estradiol. Without this hormone, the constitutively expressed transcription factor is not active. Upon addition to the culture medium, the hormone triggers the activation. We tested four ADs. B42 (79 aa) and B112 (219 aa) are short unstructured acidic peptides encoded by *Escherichia coli* genomic DNA fragments (24,25). The Gal4 AD (Gal4AD) contains the 770–881 aa fragment of the yeast transcription factor Gal4 (26). The VP16 AD is the 367–490 aa fragment of the herpes simplex virus type 1 trans-activator VP16 (27).

To analyze our four LexA-ER-AD variants, we carried out a time course of the titration in β-estradiol. To this purpose, we integrated both transcription factor and its target in the yeast genome (Supplementary Table S2). The target promoter contained four *lexA* boxes recognized by the LexA DNA-binding domain (see below) and controlled the expression of the yellow fluorescent protein Citrine (28). We monitored the activity of our LexA-ER-AD variants by measuring Citrine expression levels by flow cytometry. A yellow fluorescence signal was visible after an hour (Supplementary Figure S7). At each time point, we obtained a graded response along the β-estradiol concentration series whose maximum and steepness depended on the AD. Variants containing B42 and B112 reached their maxima after 20 h. The variants with VP16 and Gal4AD displayed peculiar induction dynamics: the maximum was reached after 5 h and the signal decreased after 20 h, depending on the β-estradiol concentration. Overall, LexA-ER-B42 was the weakest activator, and LexA-ER-VP16 and LexA-ER-Gal4AD displayed steeper titration curves than the variants containing B42 and B112.

To evaluate if these experimental observations are quantitatively consistent with the hypothesis that only characteristics of the AD variants lead to different, but predictable gene expression control, we developed a mechanistic dynamic mathematical model. The model captures, for example, protein–protein and protein–DNA interactions in detail, such that it becomes possible to address questions such as the one on consistency between synthetic constructs and their observed behaviors (see Materials and Methods for details). Specifically, we assumed that only parameters affecting the AD-RNA polymerase II interaction strength and the stability of the transcription factor protein differed between our LexA-ER-AD variants, and estimated the 45 model parameters with a (training) subset of the experimental data. The model quantitatively captured the induction dynamics in all cases (Figure 2). Overall, deviations of simulation results and experimental data were not statistically significant, neither for the training data (*P* < 10^−50^), nor for an independent validation data set that was simulated without adjusting model parameters (*P* = 1.7?10^−4^; see Materials and Methods for details); note that these statistical tests based on approximately 1400 individual data points account for the number of estimated parameters. The estimated LexA-ER-AD affinities to the general transcriptional machinery were 0.8 mM for LexA-ER-B42, 4 μM for LexA-ER-B112, 200 nM for LexA-ER-Gal4 and 27 nM for LexA-ER-VP16 (see Supplementary Table S5), well representing both the systems’ initial induction strengths and the experimentally determined affinity for the Gal4 AD alone (29). We conclude that it is possible to modulate the level of induction of the target gene with our LexA-ER-AD system by selecting the proper AD and β-estradiol concentration in a predictable manner.

### Induction and tightness of the LexA-ER-AD system

To estimate the expression range of the LexA-ER-AD system, we compared the titration curves obtained by flow cytometry with the strength of endogenous constitutive promoters. We only considered those concentration ranges in which our expression system reached steady state (from 0 to 2000 nM for LexA-ER-B42 and LexA-ER-B112; from 0 to 125 nM for LexA-ER-GalAD; from 0 to 15 nM for LexA-ER-VP16, see Figure 2). We selected a collection of frequently used promoters covering the expression range of yeast and cloned them upstream of Citrine. We assumed that the mRNA stability of all constructs was similar because the plasmids carrying the endogenous or synthetic target promoters differed only in the promoter sequence (Supplementary Table S1). To ensure similar translation efficiencies, we kept the stretch of 40 nucleotides preceding the Citrine start codon constant in all our constructs. The LexA-ER-B42 variant reached the *TEF2* promoter expression levels, while LexA-ER-Gal4AD reached the *ADH1* promoter (Figure 3A). LexA-ER-B112 and LexA-ER-VP16 reached high expression levels in the range of the*TDH3* promoter. By adjusting the β-estradiol concentration, we could obtain weaker expression levels, for example in the range of the *CYC1* or *ACT1* promoters. We conclude that the regulation of LexA-ER-AD can span the complete expression range of yeast.

To directly estimate the protein fold induction of LexA-ER-B42 and LexA-ER-B112, we performed western blotting. We prepared a dilution series of the total protein extract of the fully induced strains and probed it with an anti-GFP antibody to monitor Citrine abundance (Figure 3B). We qualitatively compared the intensity of the signal of each dilution with the one of the un-induced strain. The fold induction of LexA-ER-B42 was between 128 and 256, while the one of LexA-ER-B112 was between 200 and 400. Hence, our system has a high gene induction potential, covering the entire range of natural yeast gene expression control.

In the western blots, we observed similar Citrine levels in the un-induced strains and in the strain bearing only the target gene (and no transcription factor) (Figure 3B). To better investigate the tightness of the regulation of the four LexA-ER-AD variants, we measured their basal activity by flow cytometry. Without β-estradiol, the yellow fluorescence levels of strains with and without the transcription factor were comparable (Figure 3C), demonstrating that all four LexA-ER-AD variants were inactive without inducer.

We asked if the different induction levels of the four LexA-ER-AD variants reflected a different abundance of the transcription factor. Although all LexA-ER-AD genes were transcribed from the *ACT1* promoter (Supplementary Table S1), western blotting with an anti-LexA antibody revealed different abundances of the transcription factor variants (Supplementary Figure S8). The induction levels observed for the LexA-ER-AD variants did not directly correlate with the abundance of the transcription activator in the cells. For example, LexA-ER-Gal4AD induced moderate expression of the target gene, although it was the most abundant transcription factor. In all four variants, we detected a lower transcription factor level in the active state compared to the inactive state. This suggested a transcription-coupled degradation process (reviewed in (30)) that was also qualitatively represented by the mathematical model although this process was not enforced in the model (Supplementary Figure S9). We conclude that constitutive expression of our transcription factors covered a concentration range in which the systems were fully functional despite variable protein concentrations.

### Effects of the induction of the LexA-ER-AD system on cell physiology

To evaluate the impact of our system on cell physiology, we monitored the growth of strains containing only the synthetic transcription factor in a concentration series of β-estradiol (Figure 4A). Even at high hormone concentration (2500 nM), strains with LexA-ER-B42 or LexA-ER-B112 showed only mildly reduced growth; we therefore consider the toxic effects of these variants negligible. LexA-ER-VP16 showed strong growth inhibition when incubated with β-estradiol concentrations higher than 15 nM. This toxic phenotype explains the absence of fluorescence signal at late time points in Figure 2. We did not observe strong growth inhibition of the LexA-ER-Gal4AD strain upon incubation with concentrations up to 312 nM. The toxicity we observed in our strains depended exclusively on the activity of the transcription factor, as the growth profiles measured without β-estradiol and of the control strain without synthetic transcription factor perfectly overlapped (Supplementary Figure S10A). Also, β-estradiol alone did not reduce growth, as the growth profiles of the control strain incubated with and without the inducer were identical (Supplementary Figure S10B). In our mathematical model, we capture these phenomena as a proxy via negative feedback on RNA polymerase II abundances (see Supplementary Figure S9 for details). The simulation results were quantitatively consistent with the experimental data on dose-dependent toxicity (Figure 4B). Overall, we could prevent the toxic effects of our system by selecting the proper AD and inducer concentration.

We asked if the constitutive expression of a fluorescence reporter gene could serve as a good proxy for the physiological status of the cell. We used the red fluorescent protein mKate2 (31) expressed from the *ACT1* promoter and observed that its level was influenced by the activity of LexA-ER-GalAD and LexA-ER-VP16 (Supplementary Figure S11A). Strains containing these LexA-ER-AD variants showed increased red fluorescence, when incubated with more than 15 nM of β-estradiol. On the other hand, strains containing LexA-ER-B42 or LexA-ER-B112 displayed a constant mKate2 signal, when incubated in a concentration series of β-estradiol from 0 to 2000 nM. We analyzed the mKate2 levels in strains containing LexA-ER- Gal4AD or LexA-ER-VP16 over time (Supplementary Figure S11B). When induced with 15 nM of β-estradiol, these strains did not accumulate mKate2. However, when these strains were induced with higher β-estradiol concentrations, the mKate2 levels increased. We speculated that the increased red fluorescence, which reflected an accumulation of mKate2 within the cells, might be a consequence of the cell cycle slowdown caused by stress due to active LexA-ER-Gal4AD or LexA-ER-VP16. We conclude that the mKate2 levels are indeed a good proxy for the physiological status of the cells and thereby they simplify the experimental observation of small toxic effects.

### Induction of LexA-ER-B42 in different growth conditions

To evaluate the induction in different growth conditions, we selected the nontoxic LexA-ER-B42 variant. We first analyzed the mRNA induction with 2000 nM β-estradiol in synthetic glucose complete minimal medium (SDC) by quantitative real time PCR (qRT-PCR). A signal appeared after 15 min, the system reached half of its maximal induction level within 45 min, and steady state was reached 1 h after induction; these dynamics were well captured by the mathematical model (Supplementary Figure S3). We observed that steady state of the fluorescence signal was reached later compared to the mRNA (compare Supplementary Figures S3 and S7). This apparent discrepancy may be explained by the delay of the fluorescence signal appearance due to intermediate steps of gene expression (such as mRNA export, translation, protein folding), protein maturation and by different stability of the species considered. We decided to use flow cytometry to evaluate the induction in different culture media. To avoid metabolic side effects, we produced a protothroph strain. We integrated empty plasmids that complemented the remaining auxotrophies of the strain carrying LexA-ER-B42 and the target gene (Supplementary Table S2). Besides SDC, which supports anaerobic growth, we tested two additional conditions. A glycerol complete minimal medium (SGlyC) supported aerobic growth. A glucose minimal medium containing proline as unique nitrogen source (SDP) mimicked nitrogen limitation.

**Figure 5.**
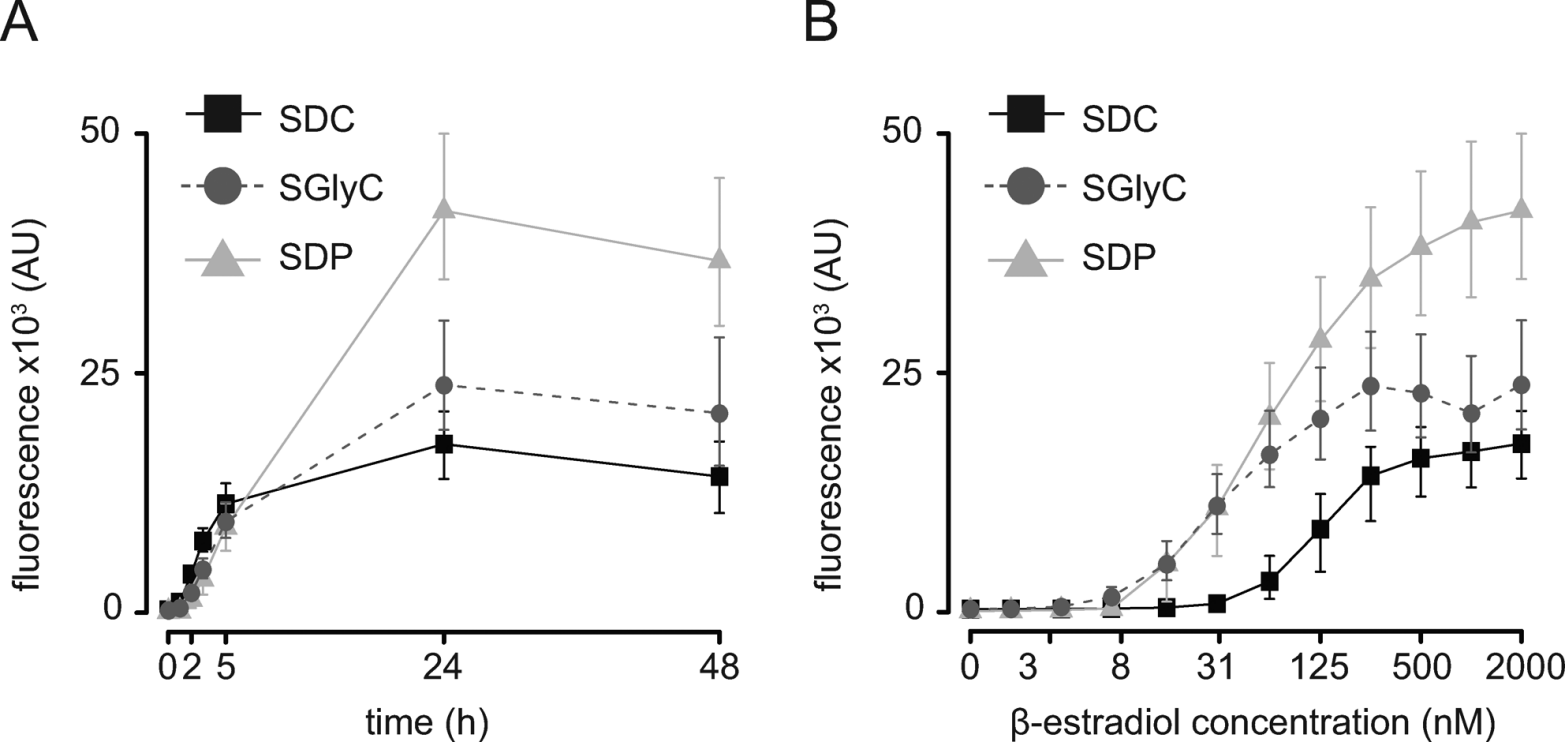
Induction of LexA-ER-B42 in different growth conditions. The induction levels of a prototroph strain containing LexA-ER-B42 and target gene with four *lexA* boxes in its promoter (FRY865) grown in SDC, SGlyC and SDP were measured by flow cytometry. Symbols represent the median and error bars the 25th and the 75th percentiles of the fluorescence signal (area) distribution measured. (**A**) Timing of the induction with 2000 nM β-estradiol. (**B**) Titration using a concentration series of β-estradiol. Cells were induced for 24 h. The x-axis is logarithmic.

Within the first 5 h, we obtained similar induction levels in the three conditions tested. However, after 24 h, cells grown in SDP showed substantially higher yellow fluorescence than those grown in SGlyC or SDC (Figure 5A) and a western blot for Citrine protein abundances confirmed the flow cytometry data (Supplementary Figure S12). To evaluate if the LexA-ER-B42 activity was titratable in the three growth conditions tested, we performed the induction in a concentration series of β-estradiol. We obtained a graded response; the maximum and the inflection point of the titration curves depended on the metabolic condition tested (Figure 5B). As observed during the time course, cells grown in SDP (or SGlyC) had a higher yellow signal than in SDC. Different specific growth rates alone cannot explain these observations because growth rates on SDP and SGlyC are very similar, and the model correspondingly overestimates induction in SGlyC due to reduced protein dilution (Supplementary Figure S4). Finally, the activity of the un-induced system did not change in SDC, SGlyC or SDP (Figure 5).

The red fluorescence connected to the mKate2 levels did not increase during the induction (Supplementary Figure S13A), confirming that our system did not cause stress in the three conditions tested. The mKate2 levels in strains grown in SGlyc or SDP were lower compared to SDC (Supplementary Figure S13). This reflected the adaptation of the activity of the *ACT1* promoter controlling mKate2 to the different growth conditions (32). As the mKate2 levels were constant along the β-estradiol concentration series (Supplementary Figure S13B), the growth conditions did not influence the range of toxicity of the active LexA-ER-B42. We conclude that our synthetic transcription factor could be induced independent of the metabolic conditions tested.

### Synthetic target promoter features

**Figure 6.**
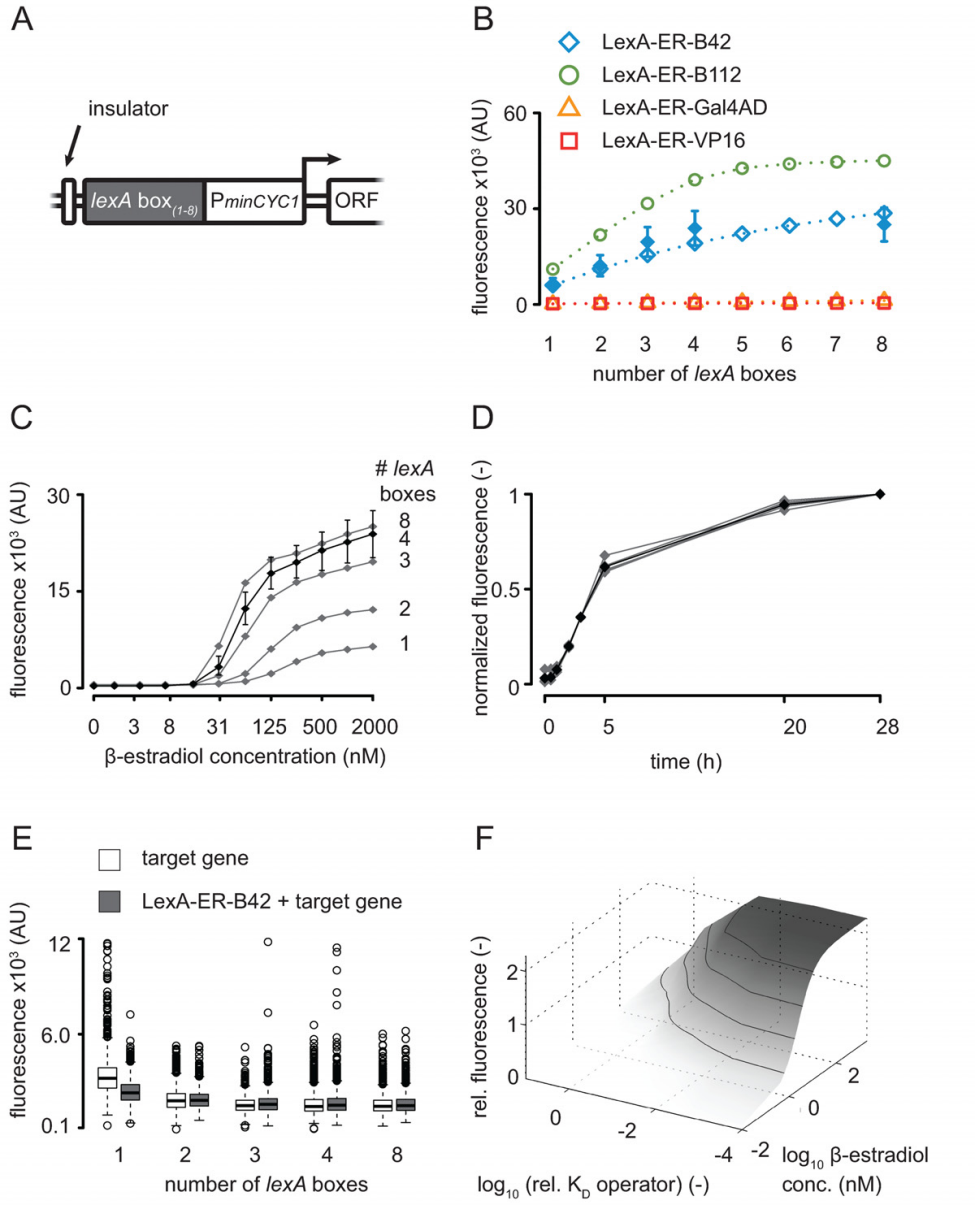
Characterization of the synthetic target promoter by flow cytometry. (**A**) Structure of the synthetic target promoter. Insulator: DEG1 terminator sequence; P*minCYC1*: minimal *CYC1* promoter; ORF: open reading frame. (**B**) Experimental (filled symbols, mean ± standard deviation) and simulated (open symbols and dashed lines) dependency of fluorescence output on the number of *lexA* boxes after 28 h induction with 2000 nM β-estradiol; all computational results, except for four *lexA* boxes are independent predictions. The strains used for this experiment are FRY400, FRY401, FRY403, FRY417 and FRY418. (**C**) Titration of the strains bearing LexA-ER-B42 and the target gene (Citrine) with one, two, three, four or eight *lexA* boxes in the promoter (FRY400, FRY401, FRY403, FRY417 and FRY418), as indicated on the right. Cells were induced for 28 h in SDC with a concentration series of β-estradiol. The x-axis is logarithmic. Symbols represent the median of the fluorescence signal (area) distribution measured. For clarity, we only plotted the error bars (the 25th and the 75th percentiles of the distributions) of the induction of the strain containing four lexA boxes (FRY418). Similar error bars were observed for the other strains. (**D**) Normalized induction of strains bearing LexA-ER-B42 and one, two, three, four or eight *lexA* boxes in the target promoter driving Citrine expression (FRY400, FRY401, FRY403, FRY417 and FRY418). The strains were induced with 2000 nM β-estradiol. The gray trajectories represent the normalized median of the induction levels of each strain; the black trajectory is the mean of the normalized medians. (**E**) Basal activity of the system incubated in SDC without β-estradiol. The target gene strains only contained the target Citrine (FRY482, FRY484, FRY485, FRY486 and FRY487); the LexA-ER-B42 + target gene strains contained both constructs (FRY400, FRY401, FRY403, FRY417 and FRY418). The copy number of *lexA* boxes in the target promoter is indicated under the boxplots, which summarize the distribution of the fluorescence signal (height) measured. (**F**) Predicted alternative configurations for the example of LexA-ER-B42. Relative fluorescence output after 24-h induction for four *lexA* boxes with modified binding affinities (dissociation constant K_D_) to the transcription factor. Values are normalized to the reference operator with 2000 nM β-estradiol induction.

LexA-ER-AD recognizes its cognate DNA-binding site in the synthetic target promoter (Figure 6A). This site, here called *lexA* box, is the fragment of the bacterial *lexA* promoter containing two SOS motifs (33,34). We constructed a collection of synthetic target promoters with one, two, three, four or eight *lexA* boxes (Supplementary Table S1). Downstream of the *lexA* boxes, we placed a core promoter derived from the previously characterized *CYC1* promoter (35). We used the *CYC1* promoter region included between −68 and +16 (+1 is the most upstream transcription initiation start site), which contains two TATA elements (at −52 and −22) and an initiation region centered in +16. The TATA element at −52 controls the +16 initiation region, while the TATA element at −22 controls initiation further downstream. In our constructs, we deleted the TATA element at −22 to exclude initiation downstream of the +16 region. We termed this core promoter minimal *CYC1* promoter. To insulate the synthetic target promoter from upstream transcriptional read through, we placed the *DEG1* termination sequence (36) in front of our synthetic transcription unit.

To evaluate the effect of the number of *lexA* boxes on the induction, we used LexA-ER-B42 and measured Citrine expression levels by flow cytometry. We observed an approximately linear relationship between the number of *lexA* boxes contained in the target promoter and the intensity of the signal (Figure 6B). Cells with two, three or four *lexA* boxes induced 2-, 3- or 4-fold more than cells with one *lexA* box. While the intensity of the signal increased with the number of *lexA* boxes, the shape of the titration curve stayed constant (Figure 6C). The correlation between intensity of the induction and the number of *lexA* boxes saturated when more than four *lexA* boxes were inserted in the target promoter. The mathematical model, which was adjusted to data using four *lexA* boxes only, predicted these relations as well as a similar relationship for LexA-ER-B112 (Figure 6B). Hence, it is possible to modulate the expression of the target gene by controlling the number of *lexA* boxes in its promoter.

We asked if the number of *lexA* boxes influenced the speed of the induction. To this purpose, we normalized the time courses obtained with one, two, three, four or eight *lexA* boxes by the respective induction levels of the last time point (28 h) (Figure 6D; un-normalized time courses are plotted in Supplementary Figure S5). The normalized induction curves showed a perfect overlap, indicating that the number of *lexA* boxes in the target promoter did not influence the speed of the induction.

To evaluate the effect of the number of *lexA* boxes on the basal activity of our promoter, we compared the fluorescence levels of un-induced strains bearing different numbers of *lexA* boxes in their target promoter. Promoters containing one or two *lexA* boxes had a slightly higher basal activity (Figure 6E). As the SOS motifs display the putative polyadenylation signal TATATA (37,38), each *lexA* box may function as a transcription terminator for the ectopic transcriptional events that ‘escaped’ the insulation by the *DEG1* terminator. We therefore speculate that the lower basal activity observed when increasing the number of *lexA* boxes could result from a stronger insulation of the target promoter.

We used the mathematical model to evaluate alternative configurations of our system. In particular, we analyzed the performance with respect to the tuning of DNA-binding domain-binding site combinations. Our model predicted that, even with four magnitude higher DNA-binding affinities, one would obtain only an approximately 2-fold increased gene expression due to nonlinear saturation effects (Figure 6F). Hence, our approach based on AD engineering could complement existing methods for the design of synthetic transcription factors. Note, however, that corresponding experimental analyses are clearly beyond the scope of the present work.

To make our induction system more accessible to the yeast community, we constructed a set of cassette plasmids for PCR-based promoter replacement (Supplementary Table S1). These plasmids contain an antibiotic resistance (*HygMX*) and the synthetic target promoter with one, two, three or four *lexA* boxes followed by a multi-cloning site where sequences encoding protein tags can be easily added (Supplementary Figure S14A). We tested the cassette containing four *lexA* boxes on *URA3*. As our strains displayed the *ura3Δ0* deletion, we first reconstituted the wild-type *locus*, and then replaced the endogenous *URA3* promoter with our cassette. We obtained a strain whose *URA3* expression was regulated by β-estradiol (Supplementary Figure S14B). During transformation, the *lexA* boxes could loop out because of their repeated sequence. We checked the transformants by colony PCR and observed that only two transformants out of 24 checked had less than four *lexA* boxes.

## DISCUSSION

We engineered the LexA-ER-AD expression system to precisely control transcription in a hormone-dependent manner in *Saccharomyces cerevisiae*. In contrast to most efforts in the field, we employed variants of ADs, and, combined with a mathematical model, we demonstrated that this design parameter offers currently under-used possibilities for engineering transcription control systems with quantitatively predictable performance. Our LexA-ER-AD is a modification of the widely used Gal4-ER-VP16, and it overcomes intrinsic limitations of Gal4-ER-VP16. A crucial result of our work is the implementation of a nontoxic transcription factor. We showed that the AD could have a role in the toxic phenotype frequently observed in synthetic expression systems such as Gal4-ER-VP16 (9). LexA-ER-B42 and LexA-ER-B112 did not have toxic effects, while the variants containing VP16 and Gal4AD did, when activated with β-estradiol.

Besides avoiding toxicity, we also wanted a system that is controllable irrespective of the cell's physiological state. We therefore substituted the Gal4 DNA-binding domain with the bacterial LexA. Theoretically, transcription factors with a heterologous DNA-binding domain do not target sites in the host genome and do not require genetic modifications of the host strain for proper activity in different physiological contexts. Reduction of the off-targets of the heterologous transcription factor also results in reduced toxicity: transcription factors based on artificial zinc-finger DNA-binding domains do not have any off-targets and they do not inhibit growth, even when they contain the VP16 AD (10). By keeping the ER, we ensured that LexA-ER-AD regulation is independent of yeast metabolism. The estrogenic hormone β-estradiol does not play a role in yeast metabolism, therefore it allows gene expression regulation without altering the nutritional composition of the culture medium (8). Moreover, the ER allows tight regulation of heterologous transcription factors (6,9).

We constructed our LexA-ER-AD system to precisely adjust the expression of the target gene to low, intermediate or high levels, rather than for overexpression. While LexA-ER-Gal4AD and LexA-ER-VP16 caused a steep graded response, the less steep induction with LexA-ER-B42 and LexA-ER-B112 allowed a finer and more precise regulation of the target gene. We classified the variants containing B112 and VP16 as strong, since their maximal induction levels mapped in the range of the *TDH3* promoter, one of the strongest promoters known in yeast (39,40). We defined the variants with B42 and Gal4AD as moderate, as their maxima were similar to the expression levels of the *ADH1* and *TEF2* promoters (40). The possibility to adjust the induction range and the steepness of the titration curve by simply exchanging the AD makes our system extremely versatile and applicable in different contexts, in contrast to other available systems that only offer steep and by consequence less controllable inductions (9,10).

The structure of the target promoter represents an additional layer for gene expression regulation. By modifying the number of transcription factor-binding sites, it is possible to adjust the strength of the induction (11,41). With this approach, we could control the induction without influencing the timing of the process or the shape of the titration curves. This allows scaling up or down the expression levels of the target gene without changing other aspects of the induction that could have unexpected effects on the process under study. Moreover, with our system it is possible to achieve the stoichiometric expression of different genes at the same time by simply adjusting the number of *lexA* boxes in each target promoter. In general, the strength of the target promoter can also be modulated by modifying the affinity of the DNA-binding domain to its target sites (10,41). However, this approach could also impact the timing of the induction or the shape of the titration curves, and it could be limited by saturation effects. By combining the optimal AD in the LexA-ER-AD with the optimal number of *lexA* boxes in the target promoter, we expect to be able to precisely control all possible levels of gene expression with our inducible system.

## SUPPLEMENTARY DATA

Supplementary Data are available at NAR Online.

SUPPLEMENTARY DATA
