# Supplementary material for: Inducible, tightly regulated and growth condition-independent transcription factor in Saccharomyces cerevisiae
Source: Nucleic Acids Res. 2014 Jul 17;42(17):e130. doi: 10.1093/nar/gku616 (PMC4176152; doi:10.1093/nar/gku616)
Supplement: SUPPLEMENTARY DATA [file supp_42_17_e130__index.html]

Inducible, tightly regulated and growth condition-independent transcription factor in Saccharomyces cerevisiae — SUPPLEMENTARY DATA 

# Inducible, tightly regulated and growth condition-independent transcription factor in *Saccharomyces cerevisiae*

## SUPPLEMENTARY DATA

**Files in this Data Supplement:**

- SUPPLEMENTARY DATA
