# Supplementary material for: Inducible, tightly regulated and growth condition-independent transcription factor in Saccharomyces cerevisiae
Source: Nucleic Acids Res. 2014 Jul 17;42(17):e130. doi: 10.1093/nar/gku616 (PMC4176152; doi:10.1093/nar/gku616)
Supplement: SUPPLEMENTARY DATA [file supp_gku616_nar-01140-met-k-2014-File008.pdf]

Online Supplementary Information

Inducible, tightly regulated, and growth condition-independent transcription factor in *Saccharomyces cerevisiae*

Diana S. M. Ottoz, Fabian Rudolf, and Joerg Stelling

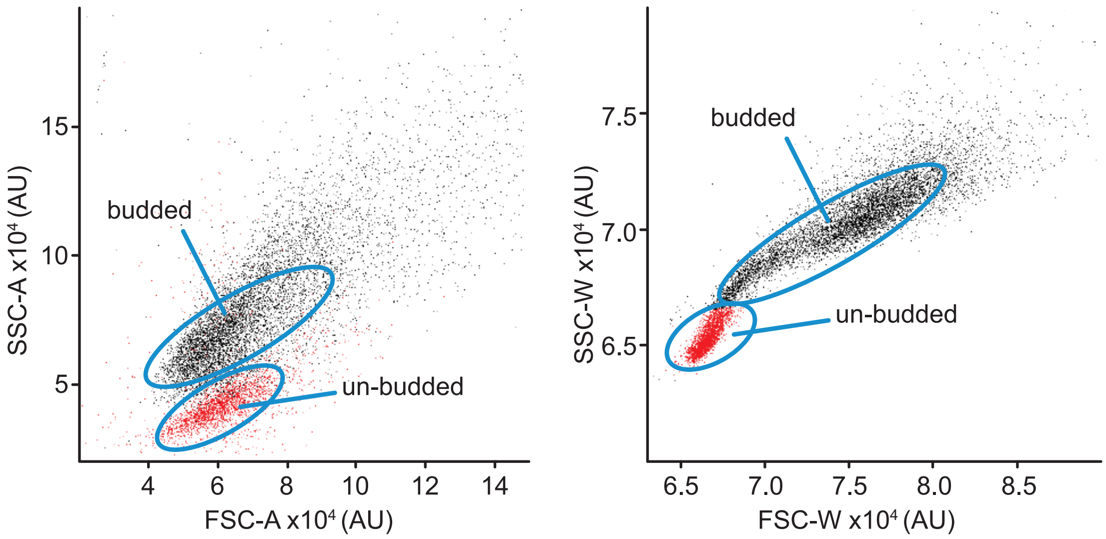

Supplementary Figure S1. Un-budded cells gating using the width forward scatter/width side scatter plots. Un-budded cells can be discriminated from budded cells in a forward scatter/side scatter (FSC/SSC) plot. Traditionally, the signal area (A) of FSC and SSC are used for gating, as depicted in the left panel. In this work, we gated un-budded cells using the signal width (W) parameter of FSC and SSC (right panel), as it results in a more precise selection. The two main populations of a yeast liquid culture (budded and un-budded cells) are highlighted with blue circles in both graphs. In red are highlighted the events we gated as un-budded. In this figure, we applied the gate on the FSC-W/SSC-W plot, and visualized the gated events also in the FSC-A/SSC-A plot.

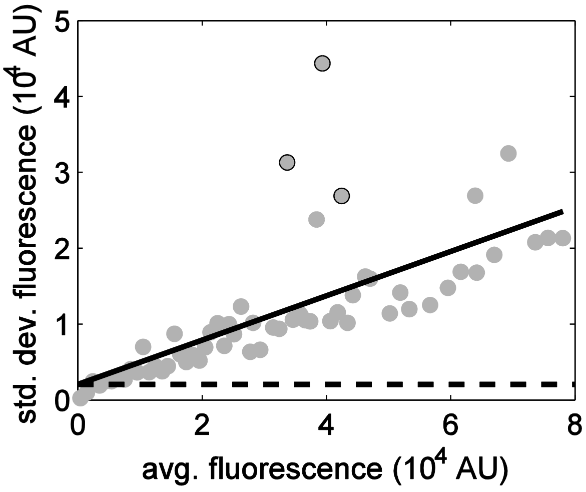

Supplementary Figure S2. Estimation of measurement variances for fluorescence readout. Fluorescence data from all experiments were pooled, binned by mean fluorescence, and standard deviation was plotted against mean fluorescence of the binned data (grey circle). Linear regression with outlier detection (outliers, black circles; regression, black solid line) identified the intercept to define the lower detection limit of the flow cytometry-based assay (dashed line; see Materials and Methods).

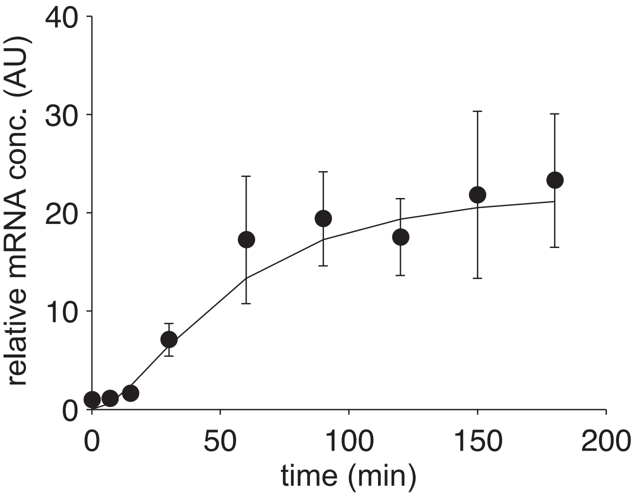

Supplementary Figure S3. Experimental (symbols, mean +/- standard deviation) and simulated (line) mRNA dynamics after induction with 2000 nM β-estradiol. We used a strain containing LexA-ER-B42 and a Citrine driven by a target promoter with four lexA boxes (FRY418). Citrine mRNA induction fold was measured by qRT-PCR, as described in the materials and methods section.

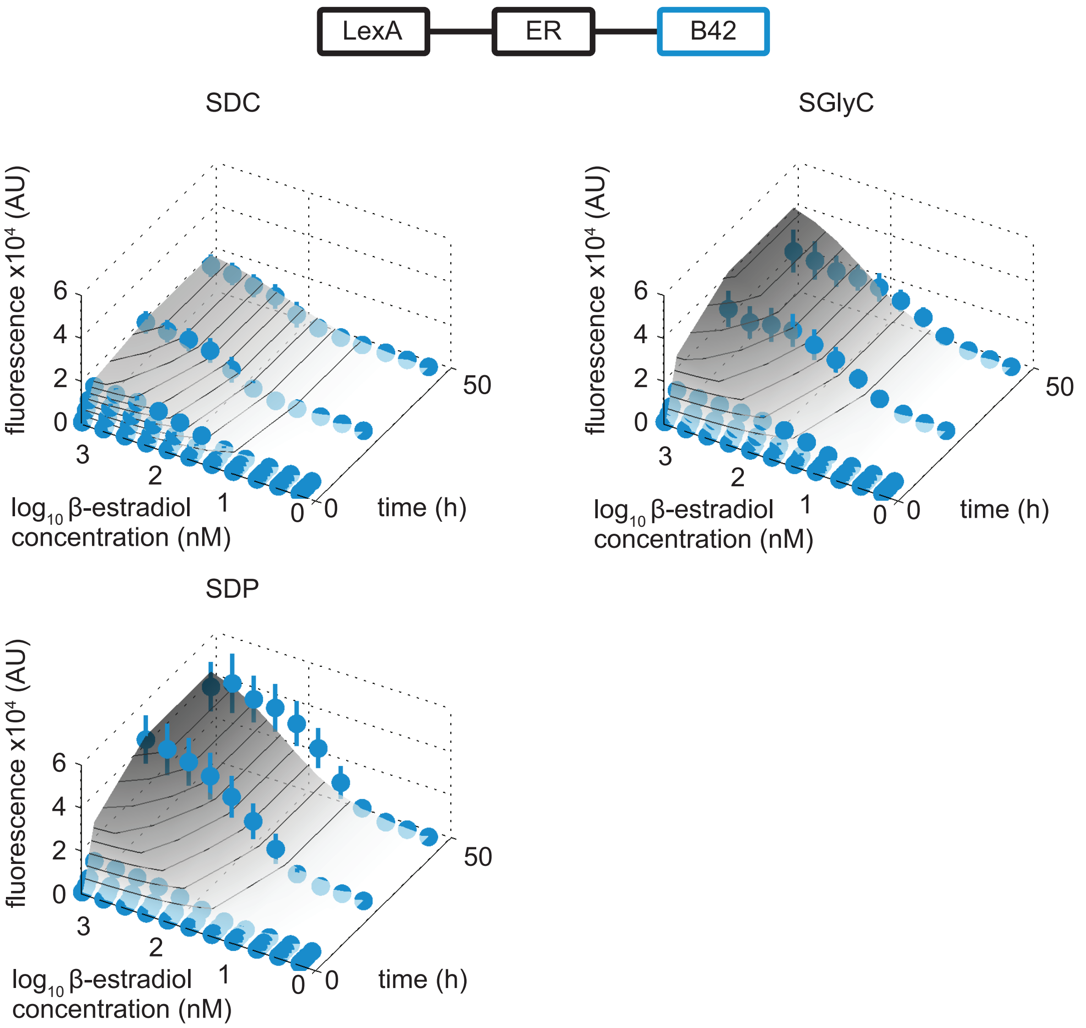

Supplementary Figure S4. Dose-response kinetics depending on growth conditions. Experimental (symbols, mean +/- standard deviation) and simulated (surfaces and contour lines) time-dependent dose-response curves for LexA-ER-B42 in SDC, SDGlyC, and SDP. Note that only the data in SDC was used for calibrating the model; the only parameters in the model reflecting growth conditions are the maximal specific growth rate (measured rate) and the translation efficiency depending on growth rate (interpolated, see Materials and Methods), which are very similar for proline and glycerol (hence, the simulation data are identical, and the model cannot capture the glycerol condition).

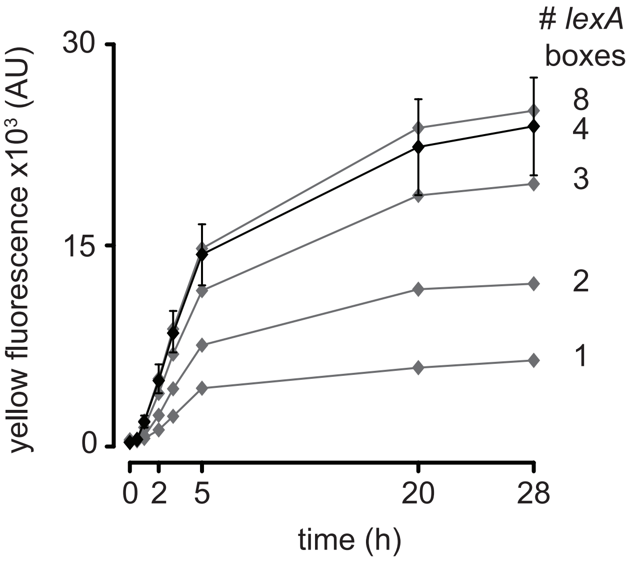

Supplementary Figure S5. Time course of the induction of strains bearing LexA-ER-B42 and the target gene (Citrine) with one, two, three, four, or eight *lexA* boxes in its promoter (FRY400, FRY401, FRY403, FRY417, and FRY418) by flow cytometry. Cells were induced with 2000 nM β-estradiol in SDC. The number of *lexA* boxes contained in the synthetic target promoter is indicated in the right side of each curve. Symbols represent the median of the fluorescence signal (area) distribution measured. For clarity, we only plotted the error bars (the 25th and the 75th percentiles of the distributions) of the induction of the strain containing four lexA boxes (FRY418). Similar error bars were observed for the other strains.

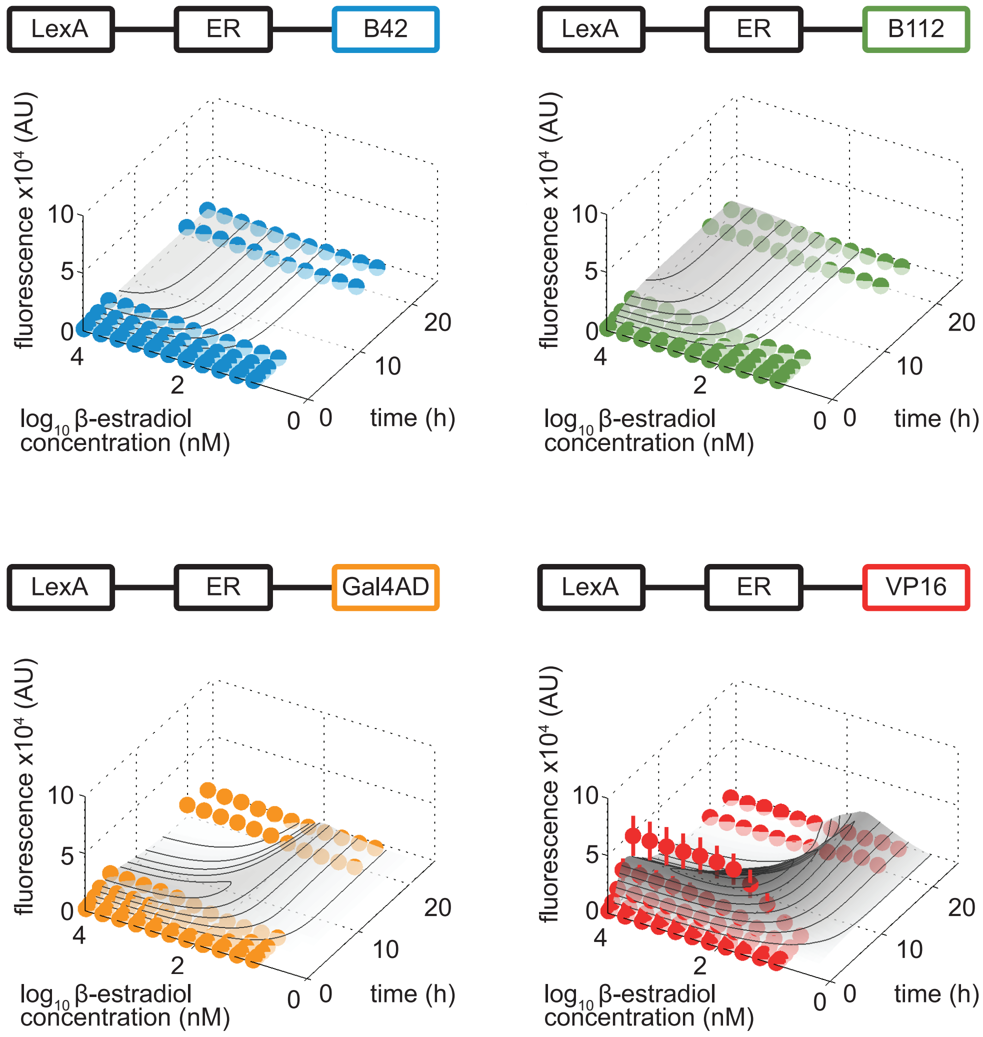

Supplementary Figure S6. Dose-response kinetics with one *lexA* box. Experimental (symbols, mean +/- standard deviation) and predicted (surfaces) data are shown as time-dependent dose-response curves analogous to Figure 2. Note that the model predictions are independent of the training data; overestimation of gene expression with LexA-ER-B112 is a manifestation of prediction errors, not of inability of the model to capture the induction dynamics in principle.

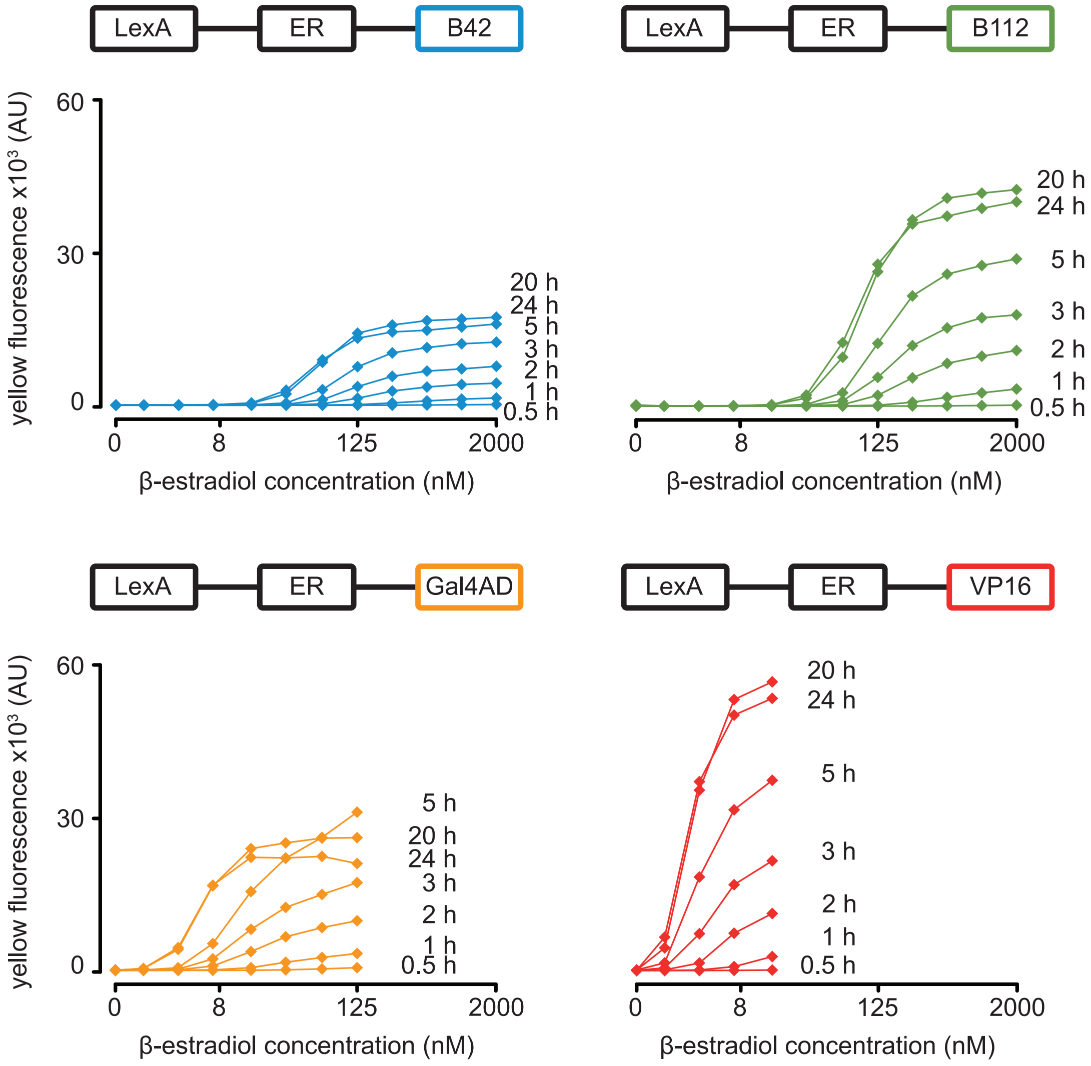

Supplementary Figure S7. Time course of the titration of LexA-ER-AD activity. Strains containing LexA-ER-B42, LexA-ER-B112, LexA-ER-Gal4AD, or LexA-ER-VP16 and a target promoter with four *lexA* boxes driving Citrine expression (FRY418, FRY666, FRY667, FRY743) were incubated in a concentration series of β-estradiol in SDC. At each time point, the induction levels were measured by flow cytometry, and plotted against the logarithmic concentration of inducer. The time point is indicated on the right side of each graph. Data points are the median of the yellow fluorescence distribution measured at each concentration of inducer.

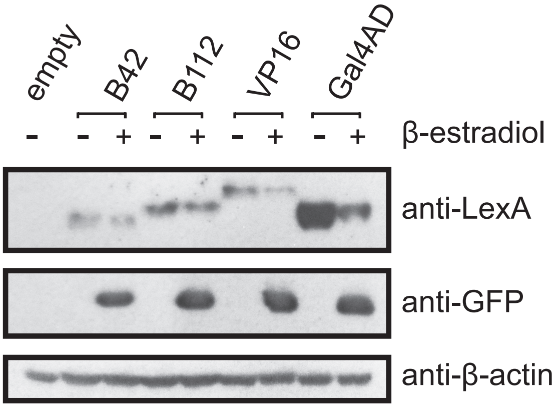

Supplementary Figure S8. Western blots of LexA-ER-AD. LexA-ER-AD and Citrine levels were assayed with anti-LexA and anti-GFP antibodies before and after 24 hours induction with β-estradiol in SDC. All strains assayed were driving the expression of Citrine under the control of four *lexA* boxes. Strains containing LexA-ER-B42 or LexA-ER-B112 (FRY418 and FRY667) were induced with 2000 nM β-estradiol. Strains containing LexA-ER-Gal4AD or LexA-ER-VP16 (FRY743 and FRY666) were induced with 62 or 15 nM β-estradiol, respectively. We used β-actin as loading control. The “empty” strain is FRY11.

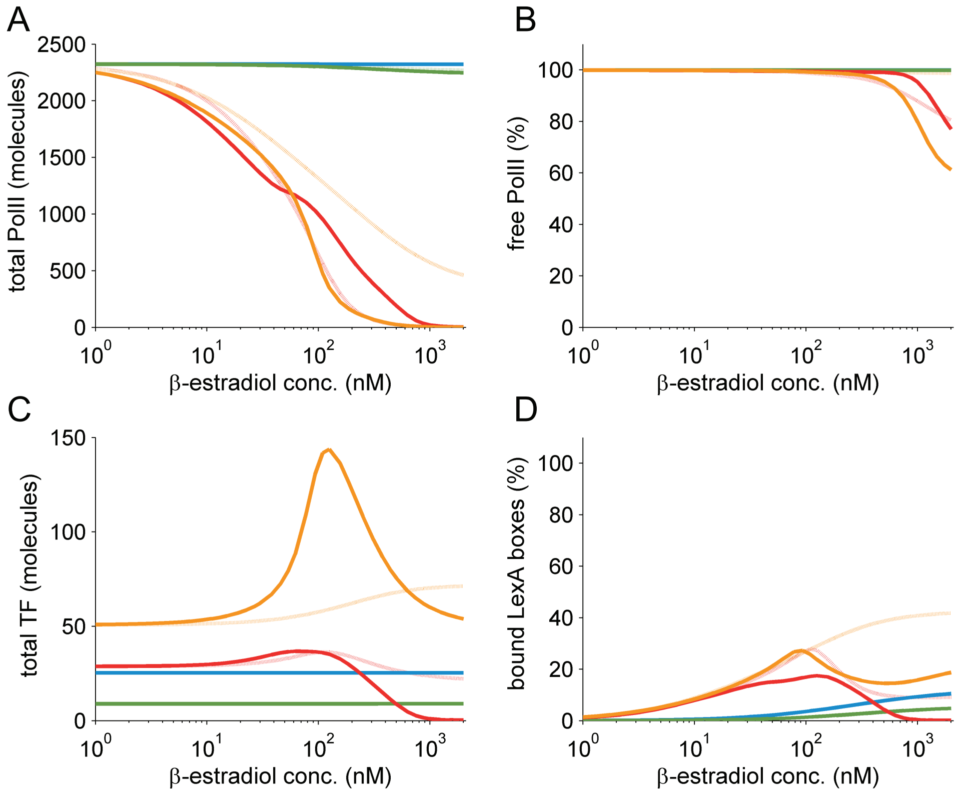

Supplementary Figure S9. Model-predicted internal system states. Total **(A)** and free **(B)** cellular concentrations of RNA polymerase II, transcription factor concentrations **(C)**, and promoter occupancy for the case of four *lexA* boxes 5h (dashed lines) and 24h (solid lines) after induction with β-estradiol. Toxicity effects are incorporated into the model by negative effects on RNA polymerase II abundances, but other possible mechanisms such as toxic effects of off-target gene induction could be incorporated in a similar feedback structure. Note that the model does not assume growth inhibition by titration of free polymerase **(B)**, and that the promoter occupancy in general is not saturated **(D)**, indicating a non-trivial influence of the activation domain characteristics on system performance.

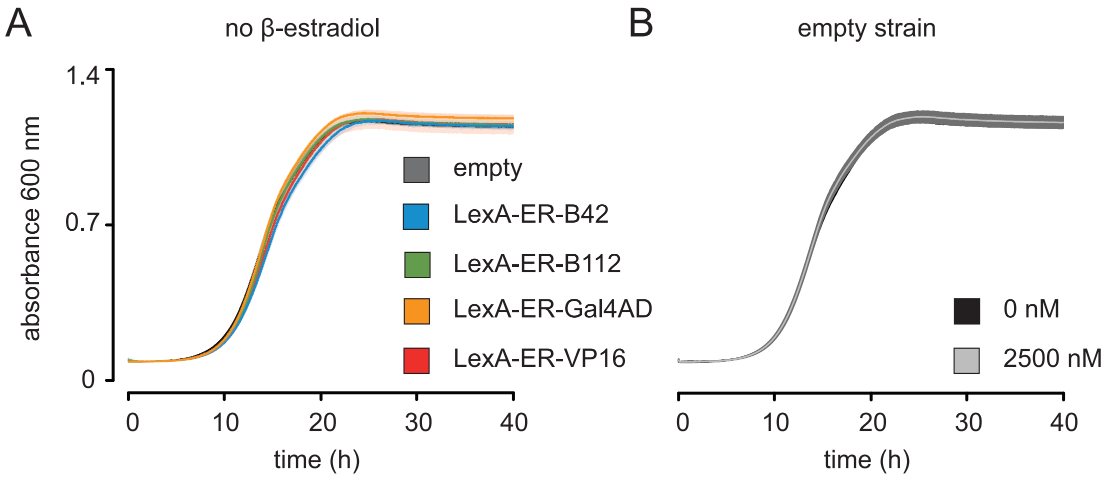

Supplementary Figure S10. Control growth profiles. For each curve, we plotted the mean of triplicates (in full color), and +/- standard deviation (in semi-transparent color). **(A)** An “empty” strain (FRY11) and strains containing only LexA-ER-AD (FRY312, FRY460, FRY544, and FRY758) were grown in SDC without β-estradiol. **(B)** The “empty” strain FRY11 was grown in SDC with and without β-estradiol.

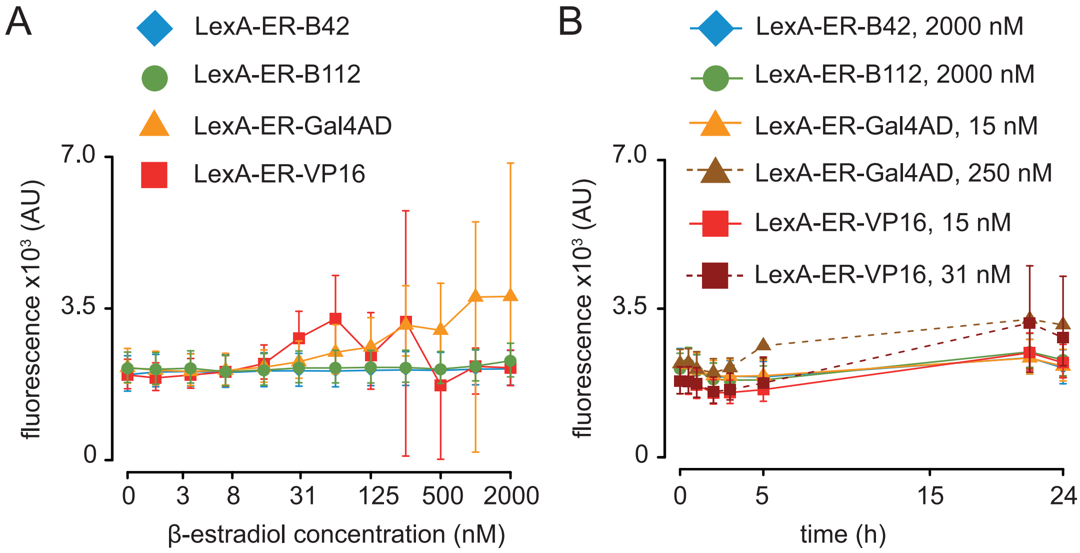

Supplementary Figure S11. Effects of the activation of LexA-ER-AD on the constitutively expressed mKate2 reporter. The mKate2 levels were monitored by flow cytometry. Symbols represent the median and error bars the 25th and the 75th percentiles of the fluorescence signal (area) distribution measured. **(A)** The red fluorescence levels in strains bearing a LexA-ER-AD variant and a target gene (Citrine) with four *lexA* boxes in its promoter (FRY418, FRY666, FRY667, and FRY743) induced with a concentration series of β-estradiol in SDC for 24 hours. The x-axis is logarithmic. **(B)** The red fluorescence levels measured in SDC over time. The amount of β-estradiol added at time 0 is indicated next to the transcription factor variant contained in each strain tested (FRY418, FRY666, FRY667, and FRY743).

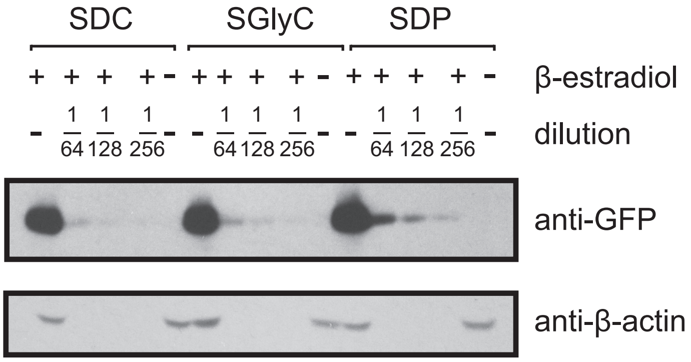

Supplementary Figure S12. Western blot to determine protein induction fold by LexA-ER-B42 upon incubation for 24 hours with 2000 nM β-estradiol in SDC, SDP, and SGlyC (strain FRY865). Citrine levels were assayed using an anti-GFP antibody. As loading control, we detected the β-actin with an anti-β-actin antibody. The induced samples were diluted as indicated. As control, we loaded the un-induced strain.

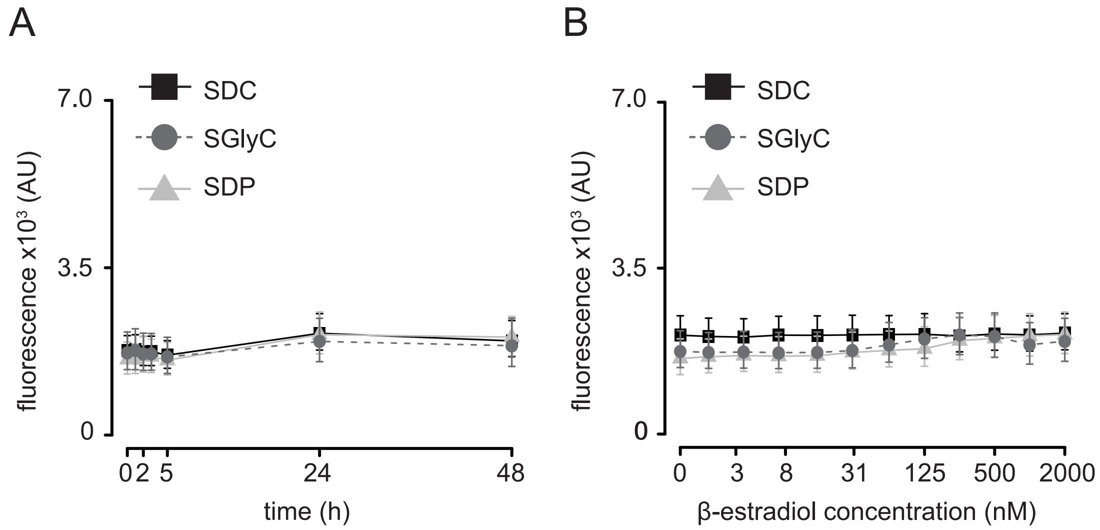

Supplementary Figure S13. Effects of the induction of LexA-ER-B42 in SDC, SGlyC, and SDP on the constitutively expressed mKate2 reporter. **(A)** The red fluorescence levels over time of a prototroph strain containing LexA-ER-B42 and target gene (Citrine) with four *lexA* boxes in its promoter (FRY865) induced with 2000 nM β-estradiol in SDC, SGlyC, and SDP. **(B)** The red fluorescence levels of the same strain incubated with a concentration series of β-estradiol in SDC, SGlyC, or SDP for 24 hours. The x-axis is logarithmic. In both panels, symbols represent the median and error bars the 25th and the 75th percentiles of the fluorescence signal (area) distribution measured.

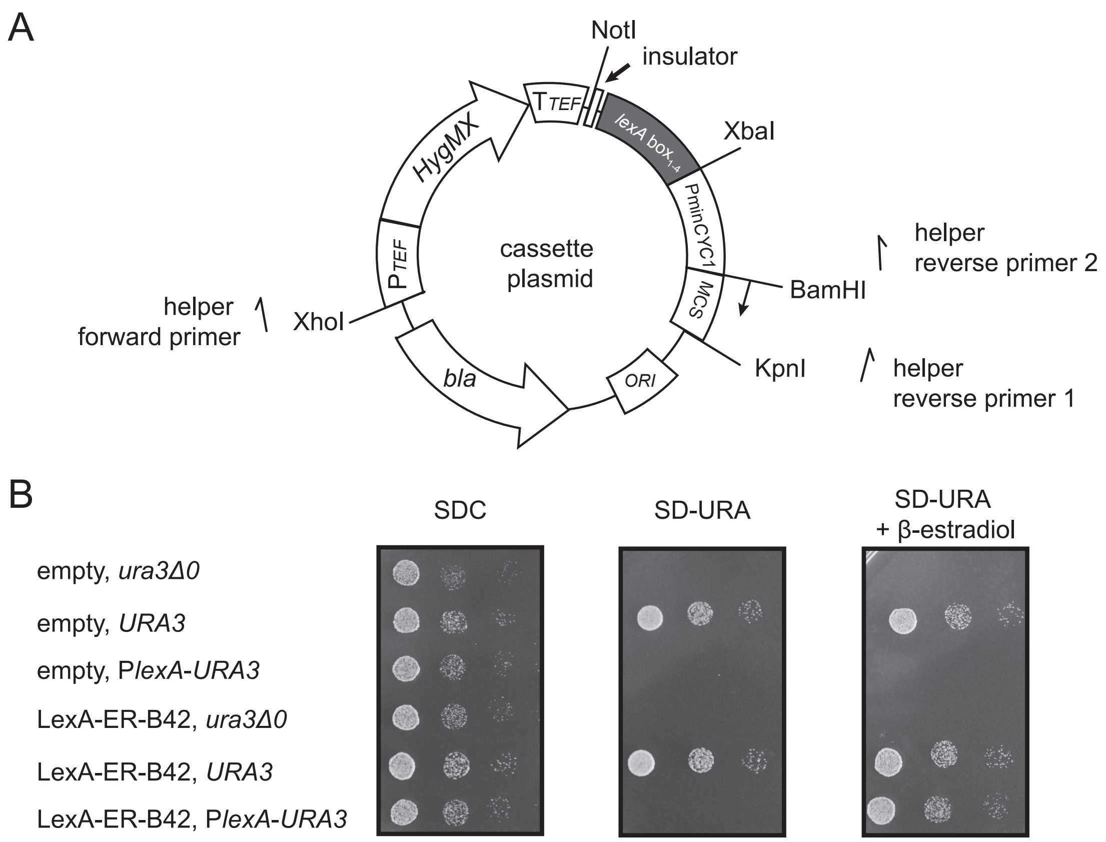

Supplementary Figure S14. (A) Map of the cassette plasmid for PCR-based primer replacement. The original backbone was pBluescript II KS(+). P: promoter; T: terminator, min*CYC1***:** minimal *CYC1*; insulator: DEG1 terminator sequence; MCS: multi-cloning site; *ORI*: origin of replication. **(B)** Spot assay to check the regulation of *URA3* by LexA-ER-B42. Cells were spotted in SDC, SD-URA, or SD-URA + 2000 nM β-estradiol. The "empty" strains do not contain LexA-ER-B42; *URA3* strains expressed *URA3* from the wild-type promoter; P*lexA-URA3* strains expressed *URA3* from our synthetic target promoter. The strains used in this figure are FRY11, FRY312, FRY1524, FRY1525, FRY1537, and FRY1538.

Supplementary Table S1. Plasmids used in this work. P: promoter; T: terminator; ER: hormone binding domain of the human estrogen receptor; ha: HA tag; insul: *DEG1* termination sequence; P*minCYC1*: minimal *CYC1* promoter; Gal4AD: Gal4 activation domain. Plasmids marked with * as well as corresponding sequences and plasmid maps are available at Addgene (www.addgene.org).

Name	Content	Yeast marker	Source
FRP229	*none*	*MET15*	this work
FRP230	*none*	*LEU2*	this work
FRP235	P*ACT1*(-1-520)-mKate2-T*ADH1*	*hisG-URA3-hisG* (to be looped out)	this work
FRP467*	P*ACT1*(-1-520)-LexA-ER-haVP16	*HIS3*	this work
FRP718*	P*ACT1*(-1-520)-LexA-ER-haB42-T*CYC1*	*HIS3*	this work
FRP791*	insul-(*lexA*-box)1-P*minCYC1*-CitrineA206K-T*CYC1*	*URA3*	this work
FRP792*	insul-(*lexA*-box)2-P*minCYC1*-CitrineA206K-T*CYC1*	*URA3*	this work
FRP793*	insul-(*lexA*-box)4-P*minCYC1*-CitrineA206K-T*CYC1*	*URA3*	this work
FRP795*	insul-(*lexA*-box)8-P*minCYC1*-CitrineA206K-T*CYC1*	*URA3*	this work
FRP800*	insul-(*lexA*-box)3-P*minCYC1*-CitrineA206K-T*CYC1*	*URA3*	this work
FRP880*	P*ACT1*(-1-520)-LexA-ER-haB112-T*CYC1*	*HIS3*	this work
FRP985	P*CYC1*(-1-287)-CitrineA206K-T*CYC1*	*URA3*	this work
FRP987	P*TDH3*(-1-680)-CitrineA206K-T*CYC1*	*URA3*	this work
FRP988	P*TEF2*(-1-402)-CitrineA206K-T*CYC1*	*URA3*	this work
FRP989	P*ADH1*(-1-1500)-CitrineA206K-T*CYC1*	*URA3*	this work
FRP929	P*ACT1*(-1-520)-CitrineA206K-T*CYC1*	*URA3*	this work
FRP991*	P*ACT1*(-1-520)-LexA-ER-Gal4AD	*HIS3*	this work
FRP1639*	P*TEF*-*HygMX*-T*TEF*-insul-(*lexA*-box)1-P*minCYC1*	*HygMX*	this work
FRP1640*	P*TEF*-*HygMX*-T*TEF*-insul-(*lexA*-box)2-P*minCYC1*	*HygMX*	this work
FRP1641*	P*TEF*-*HygMX*-T*TEF*-insul-(*lexA*-box)3-P*minCYC1*	*HygMX*	this work
FRP1642*	P*TEF*-*HygMX*-T*TEF*-insul-(*lexA*-box)4-P*minCYC1*	*HygMX*	this work

Supplementary Table S2. Yeast strains used in this work.

Name	Genotype	Source
BY4741	*MAT***a** *his3Δ1 leu2Δ0 met15Δ0 ura3Δ0*	Euroscarf
FRY11	BY4741, FRP235::T*ADH1*	this work
FRY312	FRY11, FRP718::*HIS3*	this work
FRY400	FRY11, FRP718::*HIS3*, FRP791::*URA3*	this work
FRY401	FRY11, FRP718::*HIS3*, FRP792::*URA3*	this work
FRY403	FRY11, FRP718::*HIS3*, FRP795::*URA3*	this work
FRY417	FRY11, FRP718::*HIS3*, FRP800::*URA3*	this work
FRY418	FRY11, FRP718::*HIS3* FRP793::*URA3*	this work
FRY460	FRY11, FRP467::*HIS3*	this work
FRY482	FRY11, FRP795::*URA3*	this work
FRY484	FRY11, FRP793::*URA3*	this work
FRY485	FRY11, FRP800::*URA3*	this work
FRY486	FRY11, FRP792::*URA3*	this work
FRY487	FRY11, FRP791::*URA3*	this work
FRY544	FRY11, FRP880::*HIS3*	this work
FRY666	FRY11, FRP467::*HIS3* FRP793::*URA3*	this work
FRY667	FRY11, FRP880::*HIS3*, FRP793::*URA3*	this work
FRY743	FRY11, FRP991::*HIS3*, FRP793::*URA3*	this work
FRY744	FRY11, FRP985::*URA3*	this work
FRY745	FRY11, FRP988::*URA3*	this work
FRY746	FRY11, FRP989::*URA3*	this work
FRY748	FRY11, FRP929::*URA3*	this work
FRY757	FRY11, FRY987::*URA3*	this work
FRY758	FRY11, FRP991::*HIS3*	this work
FRY865	FRY11, FRP718::*HIS3*, FRP230::*LEU2*, FRP229::*MET15*, FRP793::*URA3*	this work
FRY1524	FRY11, *URA3*	this work
FRY1525	FRY11, FRP718::*HIS3, URA3*	this work
FRY1537	FRY11, *HygMX*::insul-(*lexA*-box)4-P*minCYC1-URA3*	this work
FRY1538	FRY11, FRP718::*HIS3, HygMX*::insul-(*lexA*-box)4-P*minCYC1-URA3*	this work

Supplementary Table S3: Biochemical reaction network for the transcription factor model. Species denoted by $[X]$ and $[X \cdot Y]$ for individual components and complexes, respectively. Subscripts to species names denote the respective compartments, namely X_n and X_c for nuclear and cytoplasmic components, respectively. The uncomplexed species included in the model are: polymerase II (Pol), transcription factor (TF), hormone (H), inhibitory protein (IP), and fluorescent protein (FP), their corresponding mRNAs ($M*$), and operators / DNA sequences ($D*$). The symbol $\emptyset$ denotes a source or sink. All processes are captured by elementary chemical reations, where arrows denote the reactions and identifiers next to arrows the associated (products of) kinetic parameters. For transport processes modeled as diffusion, compartment (cytoplasm and nucleus) volumes are accounted for to establish correct mass balances.

Process	Biochemical reactions		
Expression TF	$[Pol_n]$ + $[DT_n]$	$\xrightleftharpoons[k_{D6}]{k_{A5}}$	$[Pol_n \cdot DT_n]$
”	$[Pol_n \cdot DT_n]$	$\xrightarrow{k_{P1}}$	$[MTF_c]$ + $[Pol_n]$ + $[DT_n]$
Translation TF	$[MTF_c]$	$\xrightarrow{k_{TL}}$	$[MTF_c]$ + $[TF_c]$
Degradation, TF mRNA	$[MTF_c]$	$\xrightarrow{k_{DM2}}$	$\emptyset$
TF degradation, constitutive	$[TF_c]$	$\xrightarrow{k_{DC1}}$	$\emptyset$
”	$[TF_n]$	$\xrightarrow{k_{DC1}}$	$\emptyset$
”	$[TF_c \cdot H_c]$	$\xrightarrow{k_{DC1}}$	$[H_c]$
”	$[TF_n \cdot H_n]$	$\xrightarrow{k_{DC1}}$	$[H_n]$
”	$[TF_c \cdot IP_c]$	$\xrightarrow{k_{DC1}}$	$[IP_c]$
”	$[TF_n \cdot Pol_n]$	$\xrightarrow{F_{A2} \cdot k_{DC1}}$	$[Pol_n]$
”	$[TF_n \cdot H_n \cdot Pol_n]$	$\xrightarrow{F_{A2} \cdot k_{DC1}}$	$[H_n]$ + $[Pol_n]$
”	$[TF_n \cdot DO_n]$	$\xrightarrow{F_{DC1} \cdot k_{DC1}}$	$[DO_n]$
”	$[TF_n \cdot H_n \cdot DO_n]$	$\xrightarrow{F_{DC1} \cdot k_{DC1}}$	$[H_n]$ + $[DO_n]$
Degradation active TF	$[TF_n \cdot H_n \cdot DO_n \cdot Pol_n]$	$\xrightarrow{F_{A1} \cdot F_{DC1} \cdot k_{DC1}}$	$[H_n]$ + $[DO_n]$ + $[Pol_n]$
”	$[TF_n \cdot DO_n \cdot Pol_n]$	$\xrightarrow{F_{A1} \cdot F_{DC1} \cdot k_{DC1}}$	$[DO_n]$ + $[Pol_n]$
Expression IP	$[Pol_n]$ + $[DI_n]$	$\xrightleftharpoons[k_{D7}]{k_{A5}}$	$[Pol_n \cdot DI_n]$
”	$[Pol_n \cdot DI_n]$	$\xrightarrow{k_{P7}}$	$[IP_c]$ + $[Pol_n]$ + $[DI_n]$
IP degradation, constitutive	$[IP_c]$	$\xrightarrow{k_{DC7}}$	$\emptyset$
”	$[TF_c \cdot IP_c]$	$\xrightarrow{k_{DC7}}$	$[TF_c]$
Degradation, FP mRNA	$[MFP_c]$	$\xrightarrow{k_{DM1}}$	$\emptyset$
Translation FP	$[MFP_c]$	$\xrightarrow{k_{TL}}$	$[MFP_c]$ + $[FPu_c]$
Maturation FP	$[FPu_c]$	$\xrightarrow{k_{MG}}$	$[FP_c]$
FP degradation, constitutive	$[FP_c]$	$\xrightarrow{k_{DC2}}$	$\emptyset$
”	$[FPu_c]$	$\xrightarrow{k_{DC2}}$	$\emptyset$
Expression polymerase	$[Pol_n]$ + $[DP_n]$	$\xrightleftharpoons[k_{D5}]{k_{A5}}$	$[Pol_n \cdot DP_n]$
”	$[Pol_n \cdot DP_n]$	$\xrightarrow{F_{BP3}}$	$[MPOL_c]$ + $[Pol_n]$ + $[DP_n]$
”	$[Pol_n \cdot DP_n]$	$\xrightarrow{k_{P3} \cdot \mu}$	$[MPOL_c]$ + $[Pol_n]$ + $[DP_n]$
Translation polymerase	$[MPOL_c]$	$\xrightarrow{F_{CN} \cdot k_{TL}}$	$[MPOL_c]$ + $[Pol_n]$
Degradation, Pol mRNA	$[MPOL_c]$	$\xrightarrow{k_{DM3}}$	$\emptyset$
Polymerase degradation, constitutive	$[Pol_n]$	$\xrightarrow{k_{DC3}}$	$\emptyset$
”	$[Pol_n \cdot DT_n]$	$\xrightarrow{k_{DC3}}$	$[DT_n]$

Supplementary Table S3: Biochemical reaction network for the transcription factor model (continued).

Process	Biochemical reactions		
"	$[Pol_n \cdot DI_n]$	$\xrightarrow{k_{DC3}}$	$[DI_n]$
"	$[Pol_n \cdot DP_n]$	$\xrightarrow{k_{DC3}}$	$[DP_n]$
"	$[Pol_n \cdot GO_n]$	$\xrightarrow{k_{DC3}}$	$[GO_n]$
Degradation TF-bound poymerase	$[TF_n \cdot H_n \cdot DO_n \cdot Pol_n]$	$\xrightarrow{F_{DC3} \cdot k_{DC3}}$	$[TF_n \cdot H_n \cdot DO_n]$
"	$[TF_n \cdot DO_n \cdot Pol_n]$	$\xrightarrow{F_{DC3} \cdot k_{DC3}}$	$[TF_n \cdot DO_n]$
"	$[TF_n \cdot H_n \cdot Pol_n]$	$\xrightarrow{F_{A4} \cdot F_{DC3} \cdot k_{DC3}}$	$[TF_n \cdot H_n]$
"	$[TF_n \cdot Pol_n]$	$\xrightarrow{F_{A4} \cdot F_{DC3} \cdot k_{DC3}}$	$[TF_n]$
Diffusion TF cytoplasm - nucleus	Diffusion: $[TF_c]$	$\underset{D_{TF}}{\overset{D_{TF}}{\rightleftharpoons}}$	$[TF_n]$
"	Diffusion: $[TF_c \cdot H_c]$	$\underset{D_{TF}}{\overset{D_{TF}}{\rightleftharpoons}}$	$[TF_n \cdot H_n]$
Complex TF - inhibitor protein	$[TF_c]$ + $[IP_c]$	$\underset{k_{D1}}{\overset{k_{A1}}{\rightleftharpoons}}$	$[TF_c \cdot IP_c]$
Complex TF - hormone	$[TF_c]$ + $[H_c]$	$\underset{k_{D2}}{\overset{k_{A2}}{\rightleftharpoons}}$	$[TF_c \cdot H_c]$
"	$[TF_n]$ + $[H_n]$	$\underset{k_{D2}}{\overset{k_{A2}}{\rightleftharpoons}}$	$[TF_n \cdot H_n]$
TF - DNA complexes	$[TF_n \cdot H_n]$ + $[DO_n]$	$\underset{k_{A3} \cdot k_{D3}}{\overset{k_{A3}}{\rightleftharpoons}}$	$[TF_n \cdot H_n \cdot DO_n]$
"	$[TF_n]$ + $[DO_n]$	$\underset{k_{A3} \cdot k_{D3}}{\overset{k_{A3}}{\rightleftharpoons}}$	$[TF_n \cdot DO_n]$
TF - PolII complexes	$[TF_n \cdot H_n]$ + $[Pol_n]$	$\underset{k_{D4i}}{\overset{k_{A4i}}{\rightleftharpoons}}$	$[TF_n \cdot H_n \cdot Pol_n]$
"	$[TF_n]$ + $[Pol_n]$	$\underset{k_{D4i}}{\overset{k_{A4i}}{\rightleftharpoons}}$	$[TF_n \cdot Pol_n]$
TF - DNA - PolII complexes	$[TF_n \cdot H_n \cdot DO_n]$ + $[Pol_n]$	$\underset{k_{D4i}}{\overset{N_{OP} \cdot k_{A4i}}{\rightleftharpoons}}$	$[TF_n \cdot H_n \cdot DO_n \cdot Pol_n]$
"	$[TF_n \cdot DO_n]$ + $[Pol_n]$	$\underset{k_{D4i}}{\overset{N_{OP} \cdot k_{A4i}}{\rightleftharpoons}}$	$[TF_n \cdot DO_n \cdot Pol_n]$
"	$[TF_n \cdot H_n \cdot Pol_n]$ + $[DO_n]$	$\underset{k_{A3} \cdot k_{D3}}{\overset{N_{OP} \cdot k_{A3}}{\rightleftharpoons}}$	$[TF_n \cdot H_n \cdot DO_n \cdot Pol_n]$
"	$[TF_n \cdot Pol_n]$ + $[DO_n]$	$\underset{k_{A3} \cdot k_{D3}}{\overset{N_{OP} \cdot k_{A3}}{\rightleftharpoons}}$	$[TF_n \cdot DO_n \cdot Pol_n]$
mRNA production	$[TF_n \cdot H_n \cdot DO_n \cdot Pol_n]$+ $[GO_n]$	$\xrightarrow{k_{P8}}$	$[TF_n \cdot H_n \cdot DO_n]$ + $[Pol_n \cdot GO_n]$
"	$[TF_n \cdot DO_n \cdot Pol_n]$+ $[GO_n]$	$\xrightarrow{k_{P8}}$	$[TF_n \cdot DO_n]$ + $[Pol_n \cdot GO_n]$
"	$[Pol_n \cdot GO_n]$	$\xrightarrow{k_{P2}}$	$[Pol_n]$ + $[MFP_c]$ + $[GO_n]$
Dilution by cell growth	$[TF_n]$	$\xrightarrow{\mu}$	$\emptyset$
"	$[TF_c]$	$\xrightarrow{\mu}$	$\emptyset$
"	$[TF_n \cdot H_n]$	$\xrightarrow{\mu}$	$[H_n]$
"	$[TF_c \cdot H_c]$	$\xrightarrow{\mu}$	$[H_c]$
"	$[TF_c \cdot IP_c]$	$\xrightarrow{\mu}$	$\emptyset$
"	$[TF_n \cdot Pol_n]$	$\xrightarrow{\mu}$	$\emptyset$
"	$[TF_n \cdot H_n \cdot Pol_n]$	$\xrightarrow{\mu}$	$[H_n]$
"	$[TF_n \cdot DO_n]$	$\xrightarrow{\mu}$	$[DO_n]$
"	$[TF_n \cdot H_n \cdot DO_n]$	$\xrightarrow{\mu}$	$[H_n]$ + $[DO_n]$
"	$[TF_n \cdot H_n \cdot DO_n \cdot Pol_n]$	$\xrightarrow{\mu}$	$[H_n]$ + $[DO_n]$
"	$[TF_n \cdot DO_n \cdot Pol_n]$	$\xrightarrow{\mu}$	$[DO_n]$
"	$[Pol_n]$	$\xrightarrow{\mu}$	$\emptyset$
"	$[Pol_n \cdot DT_n]$	$\xrightarrow{\mu}$	$[DT_n]$

Supplementary Table S3: Biochemical reaction network for the transcription factor model (continued).

Process	Biochemical reactions		
”	$[Pol_n \cdot DI_n]$	$\xrightarrow{\mu}$	$[DI_n]$
”	$[Pol_n \cdot DP_n]$	$\xrightarrow{\mu}$	$[DP_n]$
”	$[Pol_n \cdot GO_n]$	$\xrightarrow{\mu}$	$[GO_n]$
”	$[IP_c]$	$\xrightarrow{\mu}$	$\emptyset$
”	$[FP_c]$	$\xrightarrow{\mu}$	$\emptyset$
”	$[FPu_c]$	$\xrightarrow{\mu}$	$\emptyset$
”	$[MTF_c]$	$\xrightarrow{\mu}$	$\emptyset$
”	$[MFP_c]$	$\xrightarrow{\mu}$	$\emptyset$
”	$[MPOL_c]$	$\xrightarrow{\mu}$	$\emptyset$

Supplementary Table S4: Initial conditions. Prior to the simulation data shown, the initial conditions listed below were used to establish the system's steady-state by a long-term simulation.

State	Description	Value	Unit	Ref.
H_c	Default hormone conc.	$0.0 \cdot 10^0$	nM	Set according to experimental conditions.
DO_n	*lexA* boxes, 1 copy / nucleus	$5.5 \cdot 10^{-1}$	nM	–
TF_nDO_n	*lexA* boxes, 1 copy / nucleus	$1 \cdot 10^{-8}$	nM	Non-zero initial condition for technical reasons.
GO_n	*lexA* boxes, 1 copy / nucleus	$5.5 \cdot 10^{-1}$	nM	–
DP_n	Polymerase operators, 1 copy / nucleus	$5.5 \cdot 10^{-1}$	nM	–
DT_n	TF operators, 1 copy / nucleus	$5.5 \cdot 10^{-1}$	nM	
DI_n	Inhibitor operators, 1 copy / nucleus	$5.5 \cdot 10^{-1}$	nM	–
Pol_n	RNA polymerase, 2000 copies / nucleus	$1.1 \cdot 10^3$	nM	–
IP_c	Inhibitor, 10'000 copies / cell	$4.8 \cdot 10^2$	nM	–

Supplementary Table S5: Model parameter values and auxiliary functions. Scaling parameter were employed to map model concentrations (molar units) to experimental data (arbitrary units). Half-lives and dissociation constants are denoted by $t_{1/2}$ and K_D, respectively.

Name	Description	Parameter value		Comments
D_{TF}	Diffusion constant TF	9.55	s^{-1}	Estimated
F_{A1}	Rel. degradation active TF	0.1	–	Estimated
F_{A2}	Rel. degradation polymerase-bound TF	0.1	–	Estimated
F_{A4}	Rel. polymerase degradation in TF complexes w/o DNA	44.2	–	Estimated
F_{BP3}	Growth-dependent expression polymerase	$1.44 \cdot 10^{-4}$	–	Estimated
F_{CN}	Volume ratio cytoplasm / nucleus	11.3	–	Fixed
F_{DC1}	Rel. degradation operator-bound TF	1	–	Fixed
F_{DC3}	Rel. TF-bound polymerase degradation	10.9	–	Estimated
K_{MMU}	Michaelis-Menten constant growth function	735	nM	Estimated
N_{HMU}	Hill coefficient growth function	4.16	–	Estimated
N_{OP}	Effective number of *lexA* boxes for polymerase binding	1	–	Function (see Materials and Methods)
V_C	Volume cytoplasm	$3.4 \cdot 10^{-14}$	L cell^{-1}	Fixed
V_N	Volume nucleus	$3 \cdot 10^{-15}$	L cell^{-1}	Fixed
μ	Growth rate	$6 \cdot 10^{-5}$	s^{-1}	Function (see Materials and Methods)
k_{A1}	Association TF - inhibitor	1.12	nM^{-1}s^{-1}	Estimated
k_{A2}	Association TF - hormone	0.00936	nM^{-1}s^{-1}	Estimated
k_{A3}	Association TF - FP operator	0.00375	nM^{-1}s^{-1}	Estimated
k_{A4i}	Association TF - polymerase	0.00199	nM^{-1}s^{-1}	Estimated, LexA-ER-B42
k_{A4i}	Association TF - polymerase	0.00409	nM^{-1}s^{-1}	Estimated, LexA-ER-B112
k_{A4i}	Association TF - polymerase	0.00296	nM^{-1}s^{-1}	Estimated, LexA-ER-VP16
k_{A4i}	Association TF - polymerase	$5.64 \cdot 10^{-4}$	nM^{-1}s^{-1}	Estimated, LexA-ER-Gal4
k_{A5}	Association polymerase - operator (common constant)	0.00965	nM^{-1}s^{-1}	Estimated
k_{D1}	Dissociation TF - inhibitor	$1 \cdot 10^{-4}$	s^{-1}	Estimated (K_D = $8.96 \cdot 10^{-5}$ nM)
k_{D2}	Dissociation TF - hormone	$1 \cdot 10^{-4}$	s^{-1}	Estimated (K_D = 0.0107 nM)
k_{D3}	Dissociation TF - FP operator	15.6	s^{-1}	Estimated (K_D = $4.17 \cdot 10^3$ nM)
k_{D4i}	Dissociation TF - polymerase	$1.49 \cdot 10^3$	s^{-1}	Estimated, LexA-ER-B42 (K_D = $7.52 \cdot 10^5$ nM)
k_{D4i}	Dissociation TF - polymerase	16.1	s^{-1}	Estimated, LexA-ER-B112 (K_D = $3.93 \cdot 10^3$ nM)
k_{D4i}	Dissociation TF - polymerase	0.0796	s^{-1}	Estimated, LexA-ER-VP16 (K_D = 26.9 nM)
k_{D4i}	Dissociation TF - polymerase	0.113	s^{-1}	Estimated, LexA-ER-Gal4 (K_D = 201 nM)
k_{D5}	Dissociation polymerase - polymerase operator	24.3	s^{-1}	Estimated (K_D = $2.52 \cdot 10^3$ nM)
k_{D6}	Dissociation polymerase - TF operator	3.72	s^{-1}	Estimated
k_{D7}	Dissociation polymerase - inhibitor operator	$1 \cdot 10^{-4}$	s^{-1}	Estimated
k_{DC1}	Degradation TF	$8.98 \cdot 10^{-5}$	s^{-1}	Estimated, LexA-ER-B42 ($t_{1/2}$ = 129 min)
k_{DC1}	Degradation TF	$3.91 \cdot 10^{-4}$	s^{-1}	Estimated, LexA-ER-B112 ($t_{1/2}$ = 30 min)
k_{DC1}	Degradation TF	$7.05 \cdot 10^{-5}$	s^{-1}	Estimated, LexA-ER-VP16 ($t_{1/2}$ = 164 min)
k_{DC1}	Degradation TF	$8.02 \cdot 10^{-6}$	s^{-1}	Estimated, LexA-ER-Gal4 ($t_{1/2}$ = 1440 min)
k_{DC2}	Degradation FP	$6 \cdot 10^{-5}$	s^{-1}	Estimated ($t_{1/2}$ = 193 min)
k_{DC3}	Degradation polymerase	$6.38 \cdot 10^{-5}$	s^{-1}	Estimated ($t_{1/2}$ = 181 min)
k_{DC7}	Degradation inhibitor	$2.53 \cdot 10^{-5}$	s^{-1}	Estimated ($t_{1/2}$ = 457 min)
k_{DM1}	Degradation FP mRNA	$9.08 \cdot 10^{-4}$	s^{-1}	Estimated ($t_{1/2}$ = 13 min)
k_{DM2}	Degradation TF mRNA	0.00234	s^{-1}	Estimated ($t_{1/2}$ = 5 min)
k_{DM3}	Degradation polymerase mRNA	0.00174	s^{-1}	Estimated ($t_{1/2}$ = 7 min)
k_{MG}	Maturation rate FP	$2.89 \cdot 10^{-4}$	s^{-1}	Estimated
k_{P1}	Expression TF protein	$1.24 \cdot 10^{-5}$	s^{-1}	Estimated
k_{P2}	Expression FP mRNA	0.571	s^{-1}	Estimated
k_{P3}	Expression RNA polymerase	$1 \cdot 10^{-8}$	s^{-1}	Estimated
k_{P7}	Expression inhibitor protein	0.00665	s^{-1}	Estimated
k_{P8}	Expression FP, transition to ORF	$3.11 \cdot 10^3$	s^{-1}	Estimated
k_{TL}	Translation rate	1.06	s^{-1}	Estimated, condition-dependent (see Materials and Methods)
F_{SCFP}	Scaling parameter fluorescence	0.303	–	Estimated
F_{SCM}	Scaling parameter mRNA	1.88	–	Estimated
